# Lipid-associated macrophages reshape BAT cell identity in obesity

**DOI:** 10.1016/j.celrep.2024.114447

**Published:** 2024-07-03

**Authors:** Francesca Sciarretta, Andrea Ninni, Fabio Zaccaria, Valerio Chiurchiù, Adeline Bertola, Keaton Karlinsey, Wentong Jia, Veronica Ceci, Claudia Di Biagio, Ziyan Xu, Francesco Gaudioso, Flavia Tortolici, Marta Tiberi, Jiabi Zhang, Simone Carotti, Sihem Boudina, Paolo Grumati, Beiyan Zhou, Jonathan R. Brestoff, Stoyan Ivanov, Katia Aquilano, Daniele Lettieri-Barbato

**Affiliations:** 1 IRCCS Santa Lucia Foundation, Rome, Italy; 2 Department of Biology, University of Rome Tor Vergata, Rome, Italy; 3 Université Côte d’Azur, CNRS, LP2M, Nice, France; 4 Department of Immunology, School of Medicine, University of Connecticut, Farmington, CT, USA; 5 PhD Program in Evolutionary Biology and Ecology, Department of Biology, University of Rome Tor Vergata, Rome, Italy; 6 Laboratory of Resolution of Neuroinflammation, IRCCS Santa Lucia Foundation, Rome, Italy; 7 Department of Nutrition & Integrative Physiology, University of Utah, Salt Lake City, UT, USA; 8 Integrated Research Center (PRAAB), Campus Biomedico University of Rome, Rome, Italy; 9 Molecular Medicine Program (U2M2), University of Utah, Salt Lake City, UT, USA; 10 Telethon Institute of Genetics and Medicine, Pozzuoli, Italy; 11 Department of Clinical Medicine and Surgery, University Federico II, Naples, Italy; 12 Institute for Systems Genomics, University of Connecticut, Farmington, CT, USA; 13 Institute of Translational Pharmacology, National Research Council, Rome, Italy; 14 Department of Pathology and Immunology, Washington University School of Medicine, Saint Louis, MO, USA; 15 NOMIS Center for Immunobiology and Microbial Pathogenesis, Salk Institute for Biological Studies, La Jolla, CA 92037, USA; 16 Division of Biological Sciences, University of California, San Diego, La Jolla, CA 92093, USA; 17 IRCCS Fondazione Bietti, Rome, Italy; 18 Senior author; 19 These authors contributed equally; 20 Lead contact

## Abstract

Obesity and type 2 diabetes cause a loss in brown adipose tissue (BAT) activity, but the molecular mechanisms that drive BAT cell remodeling remain largely unexplored. Using a multilayered approach, we comprehensively mapped a reorganization in BAT cells. We uncovered a subset of macrophages as lipid-associated macrophages (LAMs), which were massively increased in genetic and dietary model of BAT expansion. LAMs participate in this scenario by capturing extracellular vesicles carrying damaged lipids and mitochondria released from metabolically stressed brown adipocytes. CD36 scavenger receptor drove LAM phenotype, and CD36-deficient LAMs were able to increase brown fat genes in adipocytes. LAMs released transforming growth factor β1 (TGF-β1), which promoted the loss of brown adipocyte identity through aldehyde dehydrogenase 1 family member A1 (Aldh1a1) induction. These findings unfold cell dynamic changes in BAT during obesity and identify LAMs as key responders to tissue metabolic stress and drivers of loss of brown adipocyte identity.

## In brief

Sciarretta et al. found obesity increases lipid-associated macrophages (LAMs) in BAT. Metabolically overloaded brown adipocytes release damaged lipids and mitochondria via EVs, cleared by BAT macrophages via CD36. EVs induce LAM identity. LAMs secrete TGF-β1, suppressing brown fat genes via Aldh1a1 induction, regulating brown-to-white fat conversion in obesity and type 2 diabetes.

## Graphical Abstract

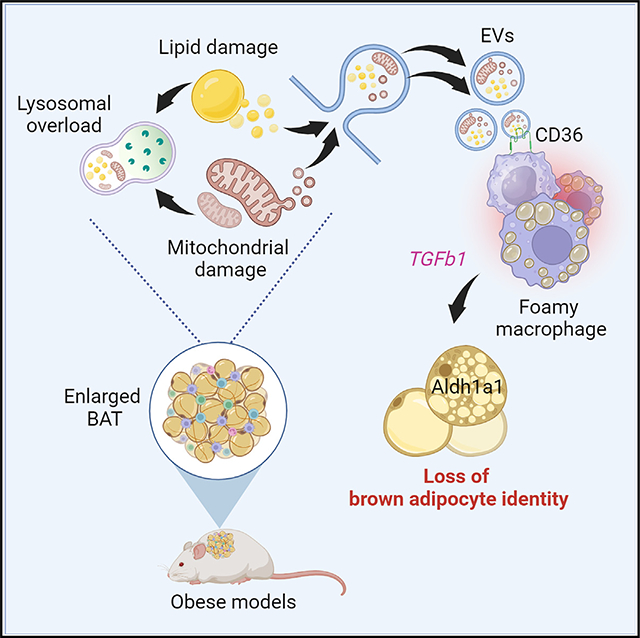

## INTRODUCTION

Type 2 diabetes (T2D) is the ninth leading cause of death globally and has quadrupled in prevalence over the previous three decades.^1^ Although pathological expansion of white adipose tissue (WAT) has been linked to the etiology of T2D,^2^ the triggering mechanisms remain not completely understood. Adipose tissue (AT) is home of an abundance of immune cells, which increase during obesity developing an immunological setting perturbing the insulin signaling.^3^ Time-resolved single-cell characterization of AT revealed that a subtype of WAT-resident macrophages called lipid-associated macrophages (LAMs) controls cell heterogeneity in the adipose niche in obesity.^4–6^

Adult humans have recently been shown to have metabolically active brown AT (BAT),^7^ which dissipates energy through the mitochondrial uncoupled respiration in the so-called non-shivering thermogenesis.^8–10^ This metabolic peculiarity led to the identification of BAT as a target to counteract T2D and obesity.^11,12^ The energy-dissipating capacity of BAT is mediated by the high content of metabolically active mitochondria, which dynamically adapt their metabolism and morphology to meet bioenergetic needs.^13,14^ During thermogenesis, adipocyte mitochondria are subject to intense metabolic stress, producing reactive oxygen species (ROS) and oxidatively damaged products that are removed by canonical autophagy processes.^10,15,16^ Accordingly, mature adipocytes with a deletion of autophagy genes (Atg3 or Atg16L1) or defective lysosomal clearance further release waste material such as dysfunctional mitochondria and lipid peroxides.^16–18^ Consistent with this, lysosomal inhibition leads to increased secretion of mitochondria in large extracellular vesicles (EVs).^19^ Recent studies showed that metabolically stressed cells, including brown adipocytes, eject damaged parts of mitochondria and macrophages participate in tissue functions by removing extracellular mitochondrial debris.^16,18,20,21^ Fate-mapping experiments in a genetic model of BAT expansion demonstrated that the BAT macrophage pool requires constant replenishment from monocytes, with subsequent development of an LAM phenotype.^22^ Although a large number of studies described the macrophage dynamics in WAT,^5,6,23,24^ a clear picture about immune cell dynamics in BAT in T2D and obesity has not been provided yet. Thus, there is an urgent need to identify the regulatory mechanisms driving disease-associated immune cell dynamics in obese BAT and to understand immune cell function in driving or protecting from obesity-related metabolic derangements.

Using a multilayered approach that integrates single-cell RNA sequencing (scRNA-seq) and deep immunophenotyping analysis of BAT of animal models of obesity, we uncovered that LAMs are responsible for the loss of brown-adipocyte identity. These results identify disease-associated LAMs that can be therapeutically targeted to restore BAT function and counteract T2D progression.

## RESULTS

### Obesity reduces brown adipocyte identity and increases LAMs in BAT

**Figure 1.**
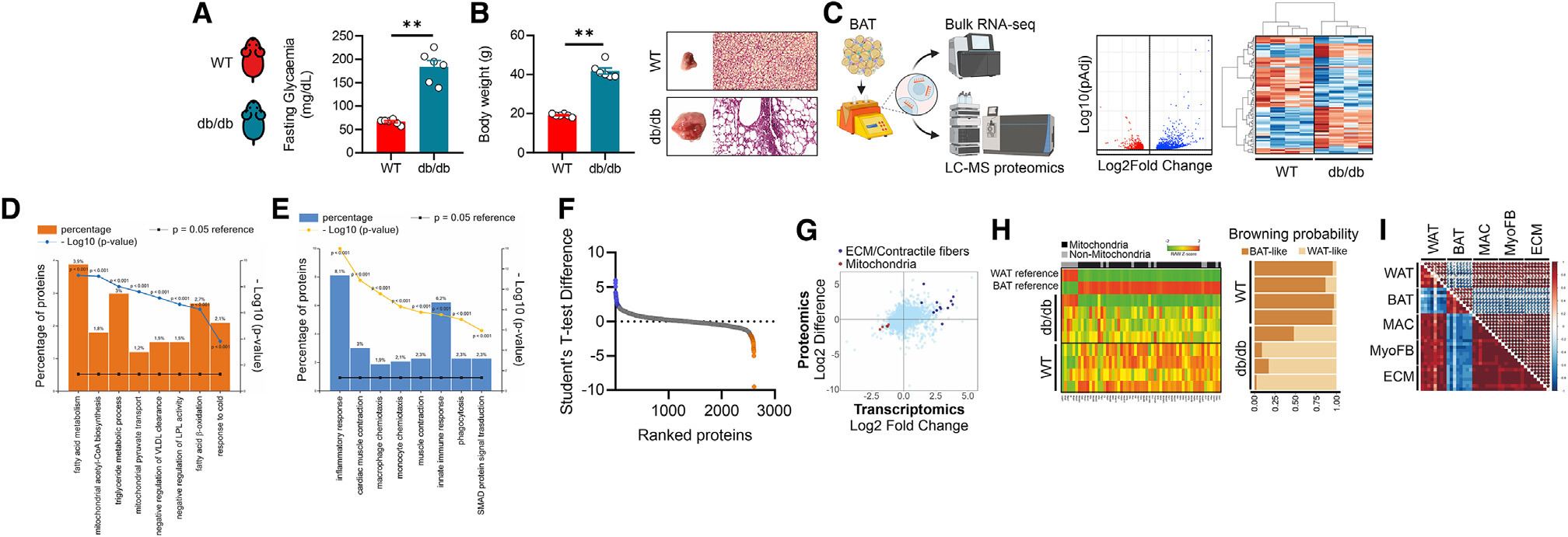
T2D induces BAT cell reshaping. (A) Fasting glycemia in 8-week-old WT and db/db mice (*n* = 6 mice/group). Data were reported as mean ± SD. Student’s t test **p < 0.01. (B) Body weight (left), representative BAT photograph and hematoxylin/eosin staining (right) in BAT of 8-week-old WT and db/db mice (*n* = 6 mice/group). Data were reported as mean ± SD. Student’s t test **p < 0.01. (C) Volcano plot and hierarchical heatmap analysis of differentially expressed genes (DEGs: pAdj < 0.05) in BAT of 8-week-old WT and db/db mice (*n* = 4 mice/group). (D and E) Functional enrichment analysis for biological processes of downregulated (D) (log2 FC < −1.5; pAdj < 0.05) and upregulated (E) (log2 FC > 1.5; pAdj < 0.05) genes in BAT of 8-week-old WT and db/db mice (*n* = 4 mice/group). (F) Differentially represented proteins (DEPs: pAdj < 0.05) identified in BAT of 8-week-old WT and db/db mice (*n* = 4 mice/group). (G) 2D plot including DEGs and DEPs in BAT of 8-week-old WT and db/db mice (*n* = 4 mice/group). (H) Heatmap (left) and browning probability (right) of DEGs analyzed by ProFAT tool^28^ (*n* = 4 mice/group). (I) Correlograms between genes pertaining to white adipocytes (WATs), brown adipocytes (BATs), macrophages (MACs), myofibroblasts (MyoFBs) and extra-cellular matrix (ECM) (*n* = 4 mice/group).

Although obesity and T2D are associated with a loss of BAT functions,^7,25^ the mechanisms leading to BAT activity failure are still unclear. To investigate this issue, we isolated BAT from 8-week-old db/db mice, which recapitulate the metabolic abnormalities characterizing T2D and obesity. db/db mice harbor a mutation in the leptin receptor gene, thus causing hyperphagia,^26^ impaired fasting glycemia (Figure 1A), and massive body weight gain accompanied by BAT expansion (Figure 1B). To explore the molecular features of BAT in db/db mice, we initially performed a bulk RNA-seq. The transcriptome profile of BAT led to the identification of 2,529 genes that were differentially expressed (adjusted *p* [pAdj] <0.05) between db/db and wild-type (WT) mice (Figure 1C). Through functional enrichment analyses, among the biological processes we found a significant downregulation of genes controlling mitochondrial fatty acid catabolism and response to cold (Figure 1D). Genes pertaining to innate immune response, monocyte/macrophage chemiotaxis, as well as muscle contraction and SMAD signal transduction were significantly induced (Figure 1E). These results recapitulated those seen in WAT of mice fed with high-fat diet (HFD).^27^ Through 2D plots we analyzed differentially expressed genes (DEGs) and proteins (Figures 1C and 1F; Table S1) in BAT of db/db mice, and an induction of extracellular matrix (ECM) and muscle contractile fibers was observed (Figure 1G); by contrast, mitochondrial components were strongly reduced (Figure 1G). To quantify the loss of brown-adipocyte identity in db/db mice, the whole-genome expression profile of BAT was analyzed by the PROFAT tool, which quantifies brown and white adipocyte content from mouse transcriptomics.^28^ In BAT of db/db mice, we observed a massive reduction in the mitochondrial brown-fat gene markers, which was consistent with lowered browning probability (Figure 1H). To investigate the molecular changes taking place specifically within mature adipocytes, we analyzed DEGs in adipocytes isolated from Ad-NuTRAP mice. This is a Cre-inducible tool mouse strain that allows labeling and simultaneous isolation of cell-type-specific nuclei and mRNA and is well suited for studying transcriptomics from specific cell types within a heterogeneous tissue.^29^ Interestingly, adipocytes from mice fed with HFD showed a loss of mitochondrial oxidative capacity and increased myofibroblast-like gene expression and sensitivity to inflammatory molecules (Figure S1A). Based on these data, we asked whether the increase in monocytes/macrophages in BAT of db/db mice was related to the loss of brown-adipocyte identity and acquirement of a myofibroblast (MyoFB)/ECM phenotype. In line with this, correlation analyses led to the identification of an inverse relationship between macrophages (MACs) and brown adipocytes, while a positive correlation was observed between MACs and MyoFB/ECM (Figure 1I). Taken together, these findings suggest a role of monocytes/macrophages in cell type reshaping in BAT of db/db mice.

**Figure 2.**
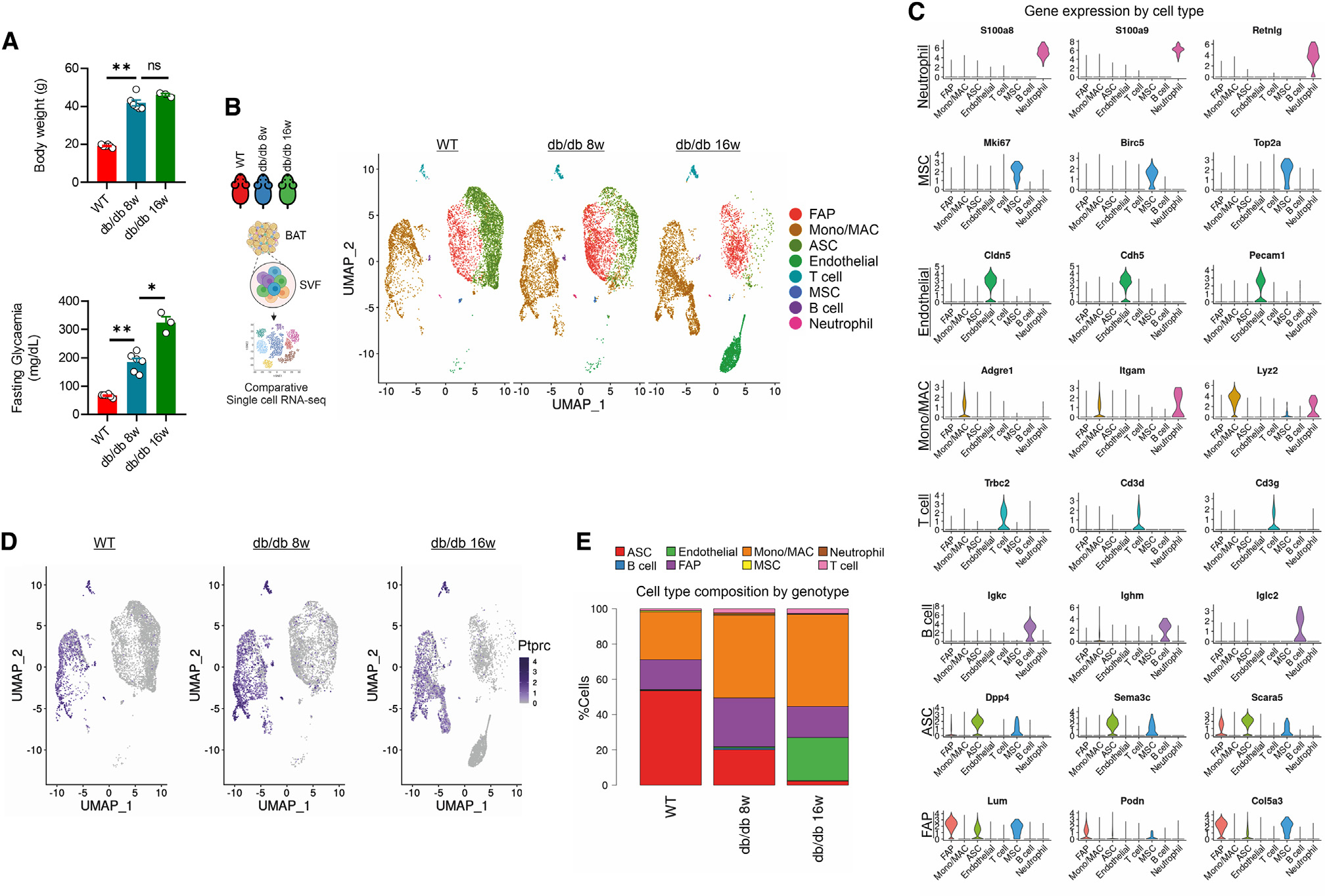
Obesity promotes monocyte/macrophage accumulation in BAT. (A) Body weight and fasting glycemia in 8- and 16-week-old WT and db/db mice (*n* = 3/6 mice/group). Data were reported as mean ± SD. Student’s t test *p < 0.05, **p < 0.01. (B) Cell clusters identified by scRNA-seq of SVFs isolated from BAT of 8- and 16-week-old WT and db/db mice (SVF pool from BAT of *n* = 3 mice/group). (C) Violin plots reporting gene markers for cell type identified by scRNA-seq of the SVFs isolated from BAT of 8- and 16-week-old WT and db/db mice (SVF pool from *n* = 3 mice/group). (D) Immune cell dynamics (Ptprc-positive cells) identified by scRNA-seq of in the SVFs isolated from BAT of 8- and 16-week-old WT) and db/db mice (SVF pool from BAT of *n* = 3 mice/group). (E) Bar plots reporting cell types identified by scRNA-seq of the SVFs isolated from BAT of 8- and 16-week-old WT and db/db mice (SVF pool from *n* = 3 mice/group).

**Figure 3.**
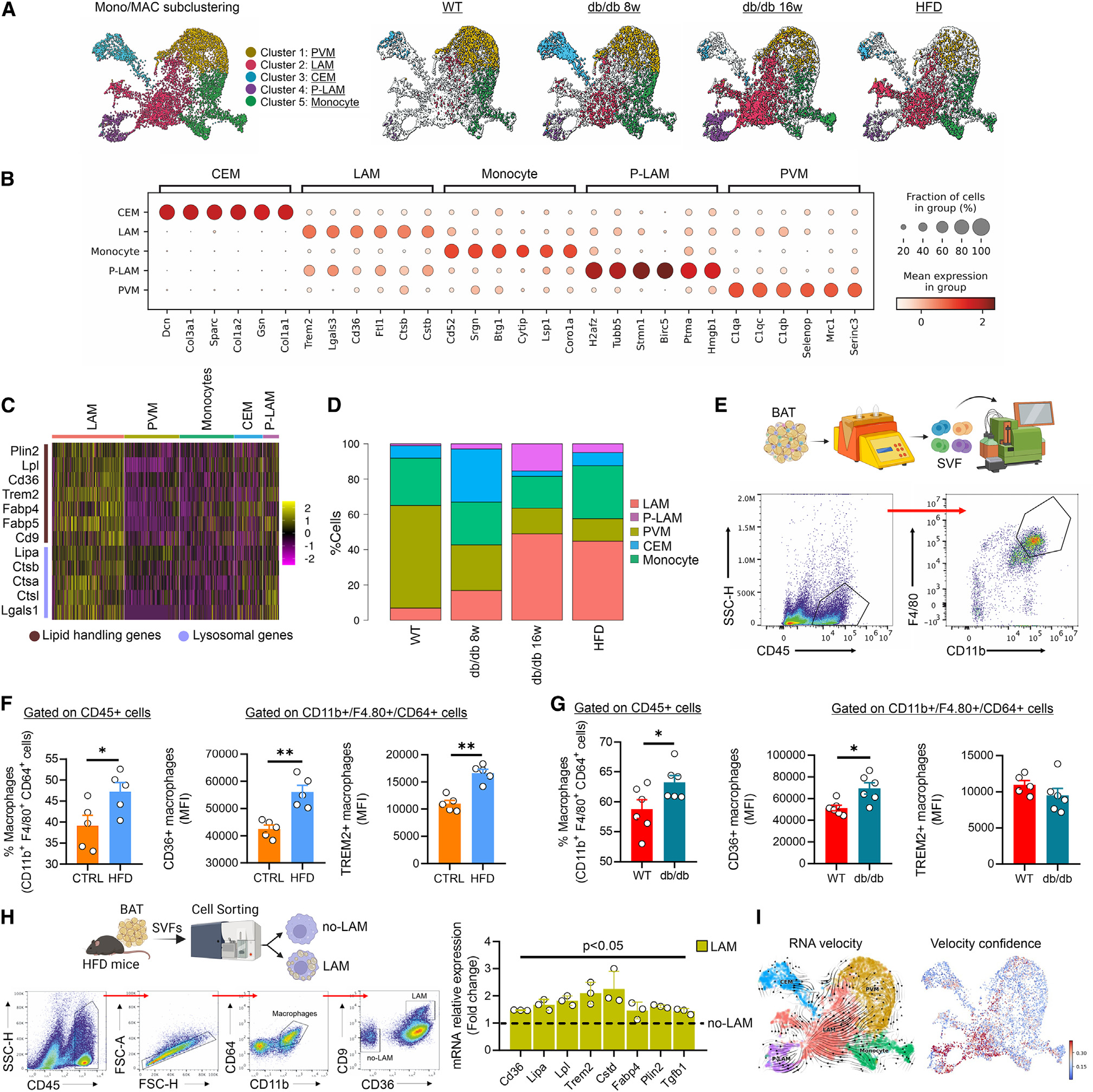
LAMs are increased in BAT of obese mice. (A) Monocyte/macrophage subclusters identified by scRNA-seq of SVFs isolated from BAT of WT, 8-, and 16-week-old db/db and HFD mice (SVF pool from BAT of *n* = 3 mice/group). (B) Dot plot reporting gene markers monocyte/macrophage subclusters identified by scRNA-seq of the SVFs isolated from BAT of WT, 8-, and 16-week-old db/db and HFD mice (SVF pool from BAT of *n* = 3 mice/group). (C) Heatmap of lipid-handling and lysosomal genes expressed in monocyte/macrophage subclusters (SVF pool from *n* = 3 mice/group). (D) Bar plots reporting monocyte/macrophage ratio identified by scRNA-seq of the SVFs isolated from BAT of WT, 8-, and 16-week-old db/db and HFD mice (SVF pool from BAT of *n* = 3 mice/group). (E–G) Gating strategy (E) of flow cytometry analyses of total macrophages (2 × 10^3^ to 10 × 10^3^) and LAM in SVFs (7 × 10^5^ to 30 × 10^4^ cells) isolated from BAT of WT, 8-, and 16-week-old HFD and db/db mice (*n* = 5/6 mice/group) (F and G). (H) Cell-sorting strategy (left) and analysis of gene expression markers (right) in LAM vs. no-LAM cells. Data were reported as mean ± SD. Student’s t test *p < 0.05, **p < 0.01. (I) Inference procedures, as employed in velocyto and scVelo, involve fitting a transcription model and predicting velocities at the single-cell level. Velocity vectors (left) and velocity confidence (right) were projected onto the UMAP.

Next, to better investigate the T2D-specific cell type dynamics in BAT, we used WT and overweight and diabetic db/db mice both at the earlier phase (8-week-old mice) and later phase of T2D (16-week-old mice) (Figure 2A). We performed a comparative scRNA-seq on BAT stromal vascular cells (SVCs) that led to the identification of a total of 21,205 quality-control (QC)-positive cells (Figure 2B). Based on the expression levels of the most variable genes, we annotated homogeneous and robust cluster of cells from scRNA-seq data (~7,000 cells per genotype), resulting in eight clusters of cells ranging from ~70 to 7,000 cells. Among these clusters, fibro-adipogenic precursors (FAPs), adipose-derived stromal cells (ASCs), endothelial cells, mesenchymal stem cells (MSCs), monocytes/macrophages (Mono/MACs), neutrophils, and T and B cells were annotated (Figures 2B, 2C, and S1B). To avoid subjectivity and to add strength to the analyses, we also performed reference-based single-cell annotation, which confirmed our broad clusters of cell populations. Of note, the average proportion of cell types in db/db mice was 48% immune cells (Figures 2D and S1C), among which about 85% were Mono/MACs (Figures 2E and S1D). These findings were consistent with the increased proportion of Mono/MACs in WAT of obese mice.^5,22^ Through the Seurat package,^30^ we next subclustered Mono/MAC cells and five different groups were identified (Figure 3A). Based on their gene expression markers, clusters 1, 3, and 5 were defined as perivascular macrophages (PVMs), collagen-expressing macrophages (CEMs), and monocytes (Figure 3A), respectively; clusters 2 and 4 were defined as LAMs and proliferating LAMs (P-LAMs) (Figures 3A and S2A), both of which characterized by high expression levels of lipid-handling and lysosomal genes (Figures 3B, 3C and S2B).^4,5^ Interestingly, clusters 2 and 4 gradually increase during T2D progression (Figures 3A and 3D). This increase was not due to LAMs deriving from infiltrating WAT as BAT LAMs in db/db mice show a peculiar enrichment of ECM and oxidative phosphorylation (OXPHOS) pathways not observed in WAT LAMs under the same condition (Figure S2C). Next, we asked whether LAM and P-LAM accumulation also occurs in dietary model of T2D. To test this, we fed adult WT mice with HFD for 10 weeks, which recapitulated some phenotypic signatures of db/db mice, including body weight gain, increased fasting glycemia, and BAT expansion (Figure S2D). Accordingly, a significant increase in LAMs and P-LAMs was observed in BAT of HFD mice (Figures 3A–3D and S2B), leading us to suppose a functional role of LAMs in BAT homeostasis. To phenotypically confirm transcriptomics data, we performed high-dimensional flow cytometry applying an established gating strategy to analyze Mono/MAC cell subsets and dynamics in BAT. In accordance with scRNA-seq data, flow cytometry revealed a significant increase in CD11b^+^/F4.80^+^/CD64^+^ monocytes/macrophages in BAT of db/db and HFD mice (Figures 3E–3G and S3A). Notably, macrophages displayed an increase in LAM markers, above all the lipid scavenger receptor CD36 (Figures 3F–3G and S3B), which was significantly reduced in the pro-inflammatory monocytes (Figure S3C). To characterize the molecular and phenotypic features of LAM, stromal vascular fraction (SVF) cells were extracted from BAT of mice subjected to HFD and sorting strategy was applied. Based on scRNA-seq, the combination of CD36 with the CD9 marker enabled us to selectively sort LAMs versus other macrophage subtypes (Figure S3D). Thus, SVF cells were sorted to obtain two distinct populations: LAM+ (CD11b^+^/CD64^+^/CD9^+^/CD36^+^) and LAM− (CD11b^+^/CD64^+^/CD9^−^/CD36^−^). Single-gene-expression analysis confirmed the characteristics of LAMs in BAT from obese mice (Figure 3H), aligning with profiling observed using scRNA-seq and flow cytometry.

The analysis of monocyte dynamics revealed that Ly6C^high^/CD62L^−^ and CCR2^−^/CD115^−^ were unchanged (Figure S3E), whereas a significant reduction of Ly6C^+^/CX3CR1^low^ pro-inflammatory monocytes were observed in obese mouse model (Figures S3F and S3G), suggesting that inflammatory monocytes transit to patrolling monocytes within BAT to differentiate into LAM. To infer the probable molecular differentiation trajectory from monocytes toward the LAM subset, we used Monocle3,^31^ a method to study the pseudo-time inference. Consistent with flow cytometry analyses, we observed a linear transition of monocytes to LAMs, with P-LAMs participating in the renewal of the LAM population in BAT of db/db mice (Figure S3H). To deeply investigate the transcription kinetics of Mono/MACs during obesity, we employed scVelo, a tool that calculates the time derivative of gene expression states between unspliced and spliced mRNAs in scRNA-seq.^32^ The uniform manifold approximation and projection (UMAP) projection revealed the landscape of Mono/MAC differentiation in BAT, highlighting that the differentiation velocity predominantly directed toward LAMs, originating from monocytes, p-LAMs, and resident macrophages (PVMs) (Figure 3I).

**Figure 4.**
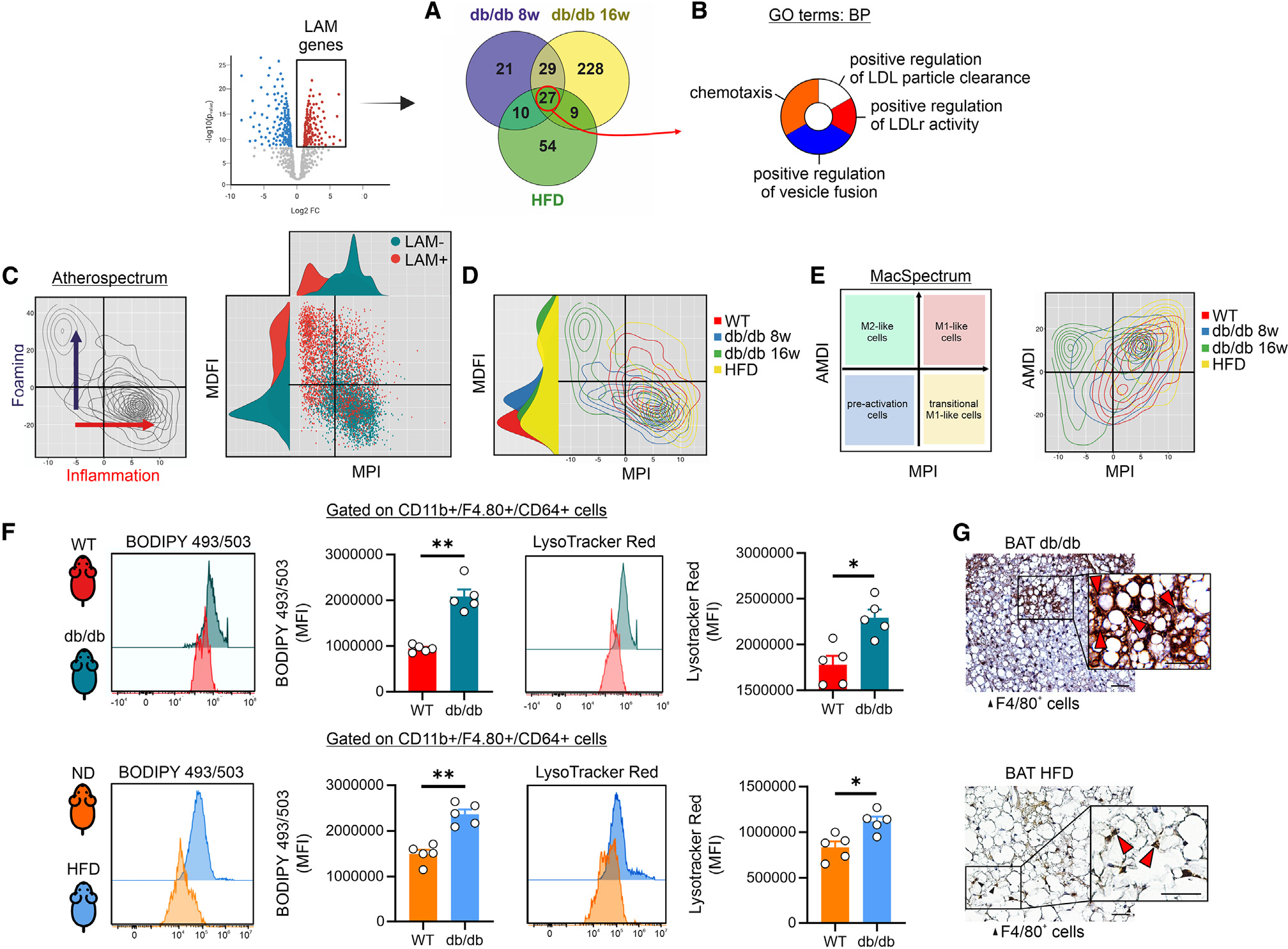
LAMs show foaming cell-like features. (A) Venn diagram of upregulated genes in LAM of WT, 8-, and 16-week-old db/db and HFD mice. (B) Functional enrichment analysis for biological processes of upregulated genes in LAM of BAT of WT, 8-, and 16-week-old db/db and HFD mice. (C) Foaming cell projection of LAM and non-LAM macrophages of BAT of WT and 16-week-old db/db mice. (D) Foaming cell projection of LAM derived from BAT of WT, 8-, and 16-week-old db/db and HFD mice. (E) Inflammatory phenotype of LAM derived from BAT of WT, 8-, and 16-week-old db/db and HFD mice. (F) Flow cytometry analyses of BODIPY 493/503 and LysoTracker red positive macrophages (CD45^+^/CD11b^+^/F4.70^+^; for gating strategy see Figure 3E) in BAT of WT and db/db mice (upper) or ND and HFD mice (lower). Data were reported as mean ± SD. Student’s t test *p < 0.05, **p < 0.01 (*n* = 5 mice/group). (G) Representative immunohistochemistry image detecting F4/80^+^ foaming-like macrophages in BAT of db/db (upper) and HFD (lower) mice. Optical magnification (O.M.) 3400, high-power field 3600. Red arrow indicates macrophages with foamy-like features. Scale bar: 20 μm.

Next, to functionally characterize gene modules of the LAMs, the upregulated genes (log2 fold change [FC] >0.85; pAdj < 0.05) in db/db and HFD mice were integrated by Venn diagram and the biological processes in the overlapping genes were analyzed (Figure 4A). The enrichment analyses revealed that LAMs in BAT take part in lipoprotein clearance and chemotaxis (Figure 4B), resembling the foam cell responses in atherosclerotic plaques.^33^ To explore this feature, we deconvolved the activation status of LAM by AtheroSpectrum, a macrophage foaming analytics tool designed to capture the heterogeneity of macrophage-derived foam cells in atherogenic foci with two indices that address key aspects of these cells: macrophage polarization index (MPI), which refers to the degree of inflammation; the macrophage-derived foam cell index (MDFI), which depicts macrophage foam cells.^34^ In accordance with their molecular phenotypes, BAT-resident LAMs showed higher MDFI and reduced MPI level if compared to macrophages negative to lipid markers (LAM−) (Figure 4C). Foaming cell features and BAT macrophage maturation were observed in BAT of both mouse models (Figure 4D) and this was in line with data obtained from the MacSpectrum annotation tool (Figure 4E), a two-index platform including MPI and the activation-induced macrophage differentiation index (AMDI).^35^ To provide evidence of foaming features in BAT macrophages, we isolated SVF cells from BAT of db/db and HFD the intracellular lipid content and lysosomal mass, indicative of foaming macrophages,^36^ was measured by BODIPY 493/503 and LysoTracker red, respectively. Subsequently, SVF cells were subjected to staining for the leukocyte marker CD45, allowing for the identification of macrophages as CD45^+^ cells co-expressing CD11b and F4/80 (Figure 3E). In line with the computation predictions, highest lipid and lysosomal content was observed in BAT macrophages of db/db (Figure 4F, upper panel) and HFD fed (Figure 4F, lower panel) mice. In line with flow cytometric measurements, immunohistochemistry analyses of BAT isolated from both db/db and HFD mice revealed an accumulation of CD206^+^ macrophages with the foaming-like feature (Figure 4G).

**Figure 5.**
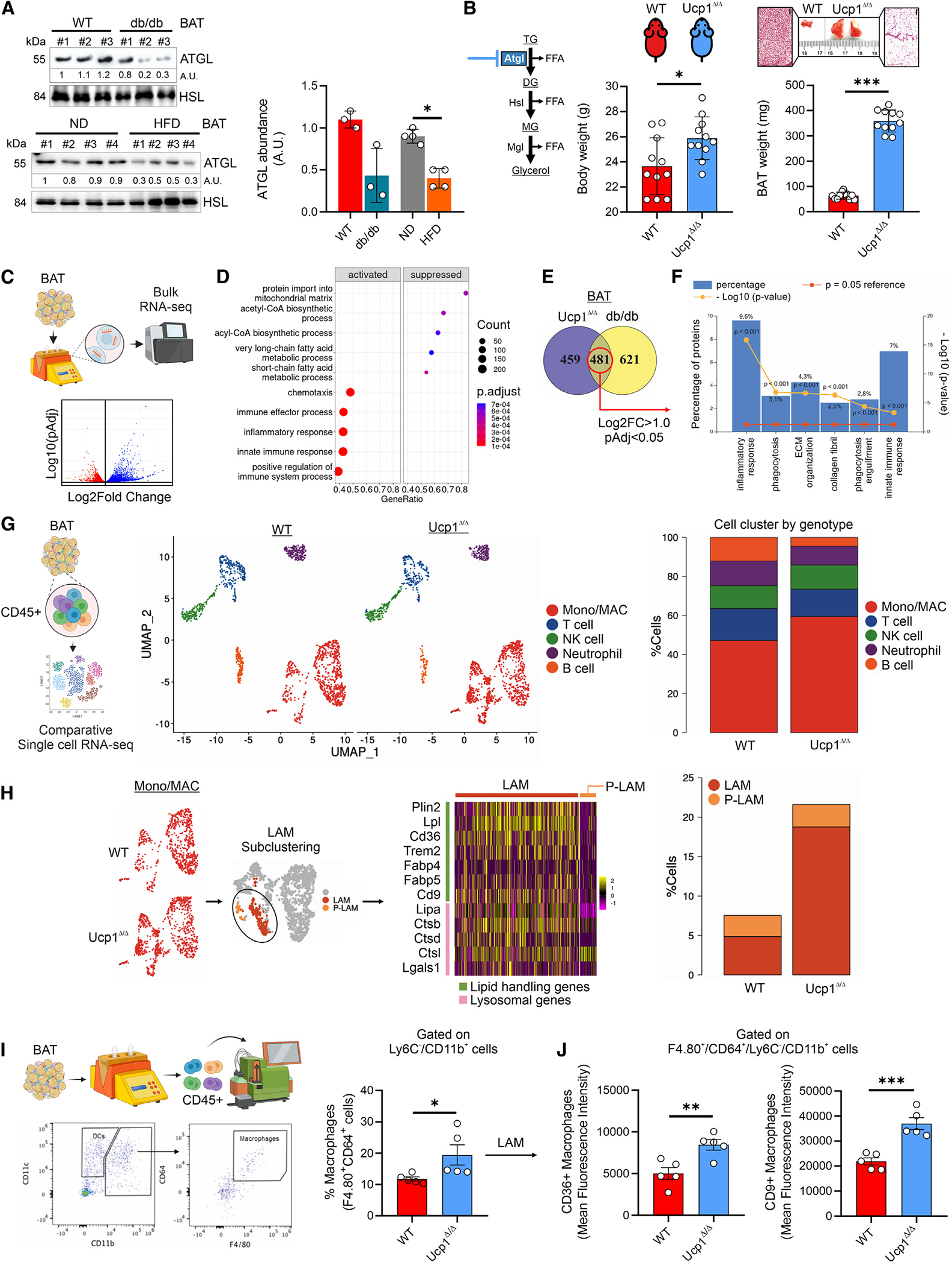
ATGL downregulation specifically in BAT promotes tissue expansion causing LAM recruitment. (A) Representative immunoblots (left) and densitometric analysis (right) of ATGL in BAT of WT, 8-week-old db/db, and HFD mice. Hormone sensitive lipase (HSL) was used as loading control. (B) Body weight (left), representative BAT photograph, hematoxylin/eosin staining (center), and BAT weight (right) of 8-week-old WT and Ucp1^Δ/Δ^ mice (*n* = 11 mice/group. Data were reported as mean ± SD. Student’s t test *p < 0.05, ***p < 0.001). (C) Volcano plot of DEGs (pAdj < 0.05) in BAT of 8-week-old WT and Ucp1^Δ/Δ^ mice (*n* = 4 mice/group). (D) Functional enrichment analysis for biological processes of downregulated (log2 FC < 1.5; pAdj < 0.05) and upregulated (log2 FC > 1.5; pAdj < 0.05) genes in BAT of 8-week-old WT and Ucp1^Δ/Δ^ mice (*n* = 4 mice/group). (E) Venn diagram of upregulated genes (log2 FC > 1.5; pAdj < 0.05) in BAT of 8-week-old WT, db/db, and Ucp1^Δ/Δ^ mice (*n* = 4 mice/group). (F) Functional enrichment analysis for biological processes of overlapping genes in BAT of 8-week-old WT, db/db, and Ucp1^Δ/Δ^ mice (*n* = 4 mice/group). (G) Cell clusters (left) and cell abundance (right) identified by scRNA-seq of Ptprc-positive cells isolated from BAT of WT and Ucp1^Δ/ΔΔ^ mice (GSE177635). (H) Heatmap of lipid-handling, lysosomal genes (left) in LAM and P-LAM and their dynamics (right) identified by scRNA-seq of Ptprc-positive cells isolated from BAT of WT and Ucp1^Δ/Δ^ mice (GSE177635). (I and J) Flow cytometry analyses of total macrophages (I) and LAM (J) in BAT of WT and Ucp1^Δ/Δ^ mice (*n* = 6 mice/group). Data were reported as mean ± SD. Student’s t test *p < 0.05, **p < 0.01, ***p < 0.001.

LAMs participate in WAT homeostasis by removing fatty acids released from metabolically stressed adipocytes.^5,6^ Additionally, an increase in adipose tissue macrophages (ATMs) was observed in obese adipose tissues, occurring independently of the fatty acid spillover from hypertrophic adipocytes.^36^ To determine whether LAM accumulation is due to fatty acids released from hypertrophic brown adipocytes, we initially examined the levels of adipose triglyceride lipase (ATGL), which is the rate-limiting enzyme in lipolysis.^37^ Interestingly, we observed a reduction in ATGL levels in the BAT of db/db and HFD mice (Figure 5A). Next, to demonstrate that LAM accumulation in expanding BAT is not contingent upon lipolysis, we employed a mouse model with selective ATGL deficiency in thermogenic adipocytes (Ucp1^Δ/Δ^). For this purpose, *Pnpla2*^flox/flox^ mice were crossed with *Ucp1*cre mice in order to ablate ATGL protein production specifically in thermogenic UCP1^+^ adipocytes following exposure. Expectedly, these mice underwent BAT enlargement similarly to db/db and HFD mice (Figure 5B), which was consistent with transcriptome profiling revealing a lower expression level (log2FC < −1.0; pAdj < 0.05) of genes controlling mitochondrial fatty acid catabolism (Figure 5C). Among the upregulated genes (log2FC > 1.0; pAdj < 0.05), we observed an enrichment of the biological processes governing innate immune response and chemotaxis (Figure 5D). Remarkably, integration of the upregulated genes (log2FC > 1.0; pAdj < 0.05) in BAT of db/db and Ucp1^Δ/Δ^ mice (Figure 5E), revealed a significant enrichment in the processes pertaining to innate immunity and phagocytosis (Figure 5F).

To investigate more in depth the immune cell dynamics in BAT of Ucp1^Δ/Δ^ mice, we subclustered scRNA-seq data from CD45^+^ cells (Figure 5G) and revealed a significant increase in Mono/MACs (Figure S4A) with a transcriptional signature referring to the LAMs (Figures 5H and S4A). To corroborate scRNA-seq data, we performed BAT immunophenotyping by high-dimensional flow cytometry. In Ucp1^Δ/Δ^ mice, we observed a low percentage of inflammatory monocytes (CD45^+^/Ly6c^high^/CX3CR1^low^/CCR2^high^) and high levels of patrolling monocytes (CD45^+^/Ly6c^low^/CX3CR1^high^/CCR2^low^) (Figure S4B). In accordance with immunological features observed in BAT of db/db and HFD mice, total BAT monocytes/macrophages (CD45^+^/F4.80^+^/CD64^+^/Ly6c^−^/CD11b^+^) (Figure 5I) as well as LAMs (CD36^+^/CD9^+^) were significantly increased in Ucp1^Δ/Δ^ mice (Figure 5J). Subsequently, brown adipocytes with downregulated Atgl (Atgl knockout [KO]) were exposed to mitochondrial stress, triggered by carbonyl cyanide 4-(trifluoromethoxy)phenylhydrazone (FCCP), and then co-cultured with bone marrow-derived macrophages (BMDMs). Significantly, we observed an increase in LAM marker (Figure S4C), prompting the hypothesis that mechanisms beyond free fatty acids might drive LAM recruitment in expanding BAT.

### LAMs control BAT homeostasis by removing extracellular mitochondrial vesicles through CD36 receptor

**Figure 6.**
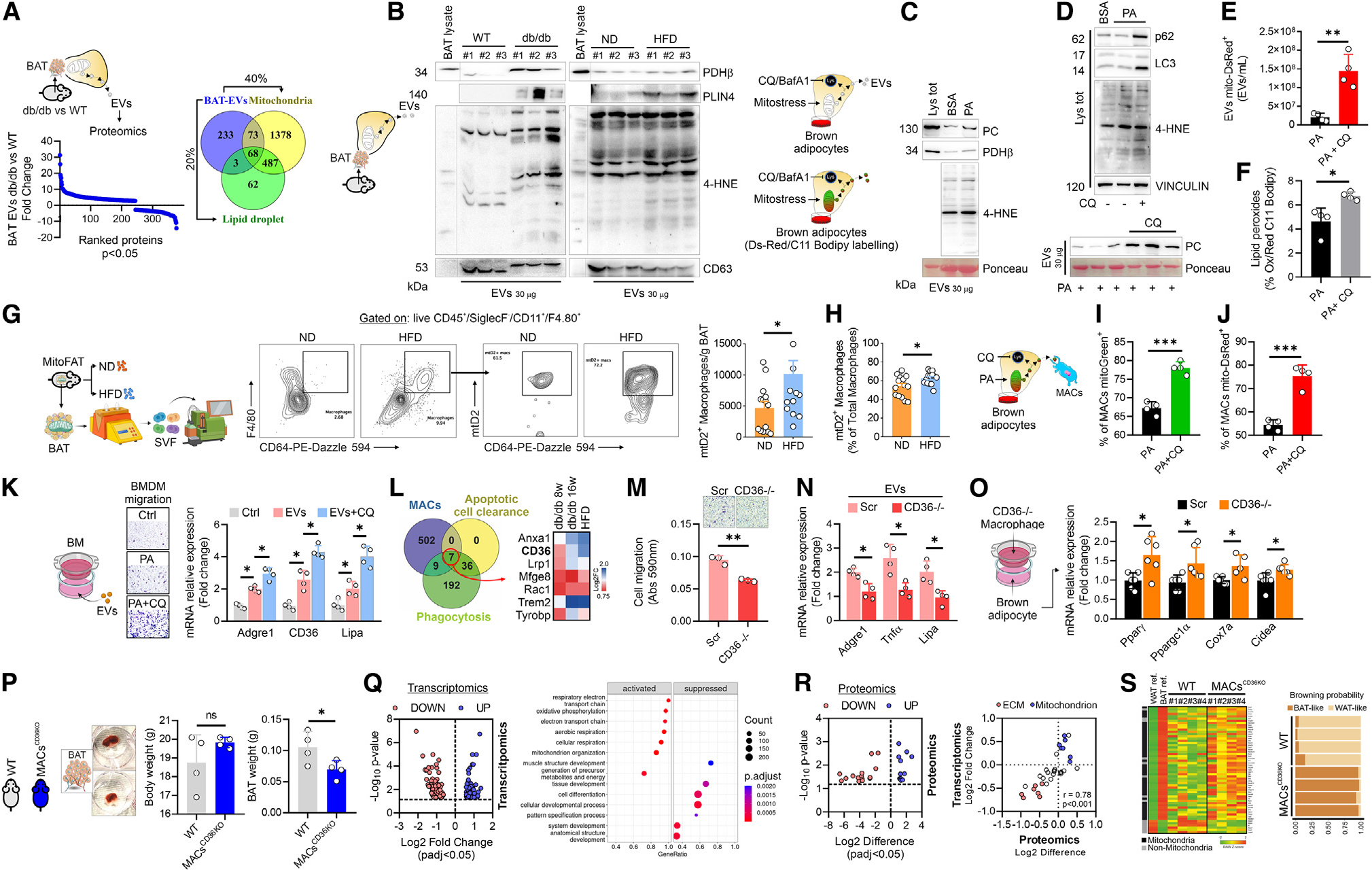
LAMs remove the extracellular vesicles released from brown adipocytes via CD36 maintaining BAT identity. (A) Proteomic profiling of EVs isolated from BAT of WT and 8-week-old db/db mice. The upregulated proteins (FC > 2.7; *p* < 0.05) were integrated with GO terms for mitochondrion (GO:0005739) and lipid droplet (GO:0005811). (B) Representative immunoblots of PDHβ, PLIN4, and 4-HNE in EVs released from BAT isolated from WT, 8-week-old db/db, and HFD mice. CD63 was used as loading control. (C) Representative immunoblots of PC, PDHβ, and 4-HNE in EVs released from palmitate (PA)-treated T37i brown adipocytes. Ponceau was used as loading control. (D) Representative immunoblots of PC, PDHβ, and 4-HNE in EVs released from palmitate (PA)-treated T37i brown adipocytes. Ponceau was used as loading control. (E) Cytofluorimetric measurements of EVs released from mitoDsRed-transfected brown adipocytes treated with PA or PA with chloroquine (CQ). Data were reported as mean ± SD. Student’s t test **p < 0.01. (F) Cytofluorimetric measurements of EVs released from Bodipy C11-loaded brown adipocytes treated with PA or PA with CQ. Lipid peroxides were expressed as oxidized-to-reduced ratio. Data were reported as mean ± SD. Student’s t test *p < 0.05. (G) Cytofluorimetric measurements of BAT macrophages positive to brown-adipocyte mitochondria in ND and HFD mice calculated for weight of tissue (*n* = 11/14 mice/group. Data were reported as mean ± SD. Student’s t test *p < 0.05). (H) Cytofluorimetric analysis of macrophages positive to brown-adipocyte mitochondria in ND and HFD mice calculated in total macrophage fraction (*n* = 14 mice/group). Data were reported as mean ± SD. Student’s t test *p < 0.05. (I and J) Flow cytometry measurements of macrophages positive to EVs released from MTG loaded (I) or mitoDsRed-transfected (J) brown adipocytes treated with PA or PA with CQ. Data were reported as mean ± SD. Student’s t test ***p < 0.001. (K) Representative image of BM chemotaxis following treatment with EVs released brown adipocytes treated with PA or PA with CQ (left). Single gene expression of macrophage markers in BM treated with brown-adipocyte EVs or brown-adipocyte EVs with CQ. Data were reported as mean ± SD. Student’s t test *p < 0.05. (L) Venn diagram of upregulated genes in BAT macrophages of 8-week-old db/db mice, GO terms of apoptotic cell clearance (GO:0043277) and phagocytosis (GO:0006909) (left), and heatmap of the seven overlapping genes in BAT macrophages of 8-week, 16-week, and HFD mice (right). (M) Scr or CD36 downregulating BM chemotaxis following brown-adipocyte EVs. Data were reported as mean ± SD. Student’s t test **p < 0.01. (N) Single gene expression of *Adgre1, Tnfα*, and *Lipa* in Scr or CD36−/− macrophages (BM) treated with brown-adipocyte EVs. Data were reported as mean ± SD. Student’s t test *p < 0.05. (O) Single gene expression of *Pparγ, Pgc1α, Cox7a*, and *Cidea* in brown adipocytes co-cultured with Scr or CD36−/− macrophages (RAW264.7 cells). Data were reported as mean ± SD. Student’s t test *p < 0.05. (P) Representative BAT photograph (left), body weight (center), and BAT weight (right) of 8-week-old WT and MAC^CD36KO^ mice (*n* = 4 mice/group). Data were reported as mean ± SD. Student’s t test *p < 0.05. (Q) Volcano plot of DEGs: pAdj < 0.05) in BAT of 8-week-old WT and MAC^CD36KO^ mice (*n* = 4 mice/group) (left). Functional enrichment analysis for biological processes of downregulated (log2 FC < −1.5; pAdj < 0.05) and upregulated (log2 FC > 1.5; pAdj < 0.05) genes in BAT of 8-week-old WT and MAC^CD36KO^ mice (*n* = 4 mice/group) (right). (R) DEPs (log2 1.5 < FC > 1.5; pAdj < 0.05) identified in BAT of 8-week-old WT and MAC^CD36KO^ mice (*n* = 4 mice/group) (left). 2D plot including DEGs and DEPs in BAT of 8-week-old WT and MAC^CD36KO^ mice (*n* = 4 mice/group) (right). (S) Heatmap (left) and browning probability (right) of DEGs analyzed by ProFAT tool^28^ (*n* = 4 mice/group).

Recent findings reported that, independently of canonical lipolysis, mature adipocytes release EVs containing lipoproteins that are actively taken up by ATMs.^16,36,38^ Mitochondrially stressed adipocytes further release EVs containing oxidatively damaged mitochondrial and lipid particles.^16,18^ To investigate the characteristics of EVs derived from the BAT of obese mice, we conducted an extensive proteomic analysis, which led to the identification of a notably high presence of mitochondrial and lipid droplet proteins in the EVs from the BAT of db/db mice (Figure 6A; Table S2). The accumulation of lipid peroxidation markers, specifically 4-HNE protein adducts, in conjunction with the elevated levels of mitochondrial and lipid droplet-associated proteins was confirmed through immunoblotting techniques (Figure 6B). To reproduce an *in vitro* model of metabolic overload, brown adipocytes were treated with palmitic acid (PA), which was effective in augmenting the production of mitochondrial ROS and lysosomal mass (Figure S5A). As in BAT of db/db and HFD mice, in brown adipocytes, PA caused the release into EVs of mitochondrial proteins and 4-HNE protein adducts (Figure 6C). Next, to demonstrate that extracellular mitochondria ejection was part of a quality-control system of this organelle, we inhibited lysosomal activity by chloroquine (CQ). Consistent with prior reports,^16,18^ lysosomal inhibition increased 4-HNE protein adducts and mitochondrial pyruvate carboxylase (Pc) in EVs (Figure 6D). Expectedly CQ treatment further increased lysosomal mass and caused an enhancement of mitochondrial ROS and lipid peroxides production in PA-treated brown adipocytes (Figure S5A). To better analyze the release of mitochondrial components and lipid peroxides, brown-adipocyte mitochondria and lipid droplets (LDs) were labeled with mitoDsRed and C11 BODIPY, respectively. EVs were collected following CQ treatment and flow cytometry measurements were carried out. Lysosomal inhibition induced a significant increase in the release of EVs positive to mitoDsRed (Figure 6E) as well as lipid peroxides (Figure 6F). To further determine whether EV release was part of a mitochondrial quality control system, we treated brown adipocytes with sublethal doses of the mitochondrial stressors FCCP or antimycin A (AA). As expected, both FCCP and AA promoted mitochondrial ejection from brown adipocytes (Figure S5B) and this process was enhanced when lysosomal clearance was inhibited by CQ or bafilomycinA1 (bafA1) (Figure S5C).

To investigate if HFD forces mitochondrial transfer from brown adipocytes to macrophages *in vivo*, we used adipocyte-specific mitochondria reporter (MitoFat) mice.^23^ Consistent with a prior study,^39^ we observed an increased level of total macrophages in BAT of HFD mice (Figure 6G). Nicely, HFD impinged mitochondrial transfer from brown adipocytes to macrophages, as demonstrated by higher percentage of mtD2+ macrophages in BAT of HFD than mice fed with normal diet (ND) (Figure 6H). To demonstrate if EVs deliver mitochondria to macrophages, we labeled adipose cells with mitotracker Green (MTG) and the released EVs (EVs^MTG+^) were used to treat macrophages. Interestingly, we observed a high percentage of EVs^MTG^-positive macrophages (Figure 6I), suggesting their capacity to phagocytize EVs from brown adipocytes. To rule out potential artifact due to labeling with MTG probe, we next used EVs released from mitoDsRed^+^ brown adipocytes (EVs^mitoDsRed^). Remarkably, we observed a significant uptake of EVs^mitoDsRed^ by macrophages and the percentage of mitochondrial transfer was increased when lysosomal activity of brown adipocytes was inhibited (Figure 6J).

Next, we sought to investigate whether EVs facilitate macrophage migration and maturation toward an LAM phenotype. To test this, we cultured bone marrow (BM) cells with EVs and observed that the highest migration capacity was accompanied by the increase of mature macrophage markers including lipidhandling genes (Figure 6K). To explore the mechanisms governing EV clearance by macrophages, we integrated the upregulated genes in BAT macrophages of db/db and HFD mice with Gene Ontology (GO) terms for phagocytosis (GO:0006909) and efferocytosis (GO:0043277). Through this approach, we identified seven overlapping genes, including CD36 scavenger receptor (Figure 6L), which was shown participate in apoptotic cell clearance (also known as efferocytosis) and EV clearance.^16,40–42^ Accordingly, CD36 downregulation in RAW264.7 macrophage cell lines (RAW264.7^CD36−/−^) diminished cell migration as well as macrophage maturation following EV treatment (Figures 6M and 6N).

The significant increase of CD36^+^ macrophages in BAT of db/db, Ucp1^Δ/Δ^, and HFD mice (Figure S6A) led us to suppose that CD36^+^ macrophages participate in the molecular loss of BAT identity. To explore this possibility, we initially co-cultured brown adipocytes with macrophages downregulating CD36 and, as reported in Figure 6O, increased expression levels of browning genes were observed. Next, we generated germline KO (CD36^−/−^) and myeloid-specific-deficiency (CD36^flox/flox^ × Csf1r^Cre^) mouse models. Interestingly, although adult mice with specific ablation of CD36 in macrophages (MACs^CD36KO^) showed no modulation in the total body weight, a significant reduction in BAT mass was observed (Figure 6P). Next, we profiled the transcriptome of BAT from MACs^CD36KO^ mice, which revealed a significant upregulation of genes pertaining to mitochondrial oxidative function (Figure 6Q); by contrast, genes controlling muscle cell and ECM were diminished (Figure 6Q). Transcriptomics data were next integrated with proteomics data and, by using a 2D plot, we demonstrated that BAT from MACs^CD36KO^ mice was enriched in mitochondrial component rather than in muscular and ECM components (Figure 6R; Table S3). Remarkably BAT of MACs^CD36KO^ mice showed increased mitochondrial BAT markers and higher browning probability compared with WT mice (Figure 6S). These results highlight the competence of LAMs to control brown-adipocyte identity.

### LAMs depleted mitochondrial content of brown adipocytes via Tgfβ1-Aldh1a1 pathway

**Figure 7.**
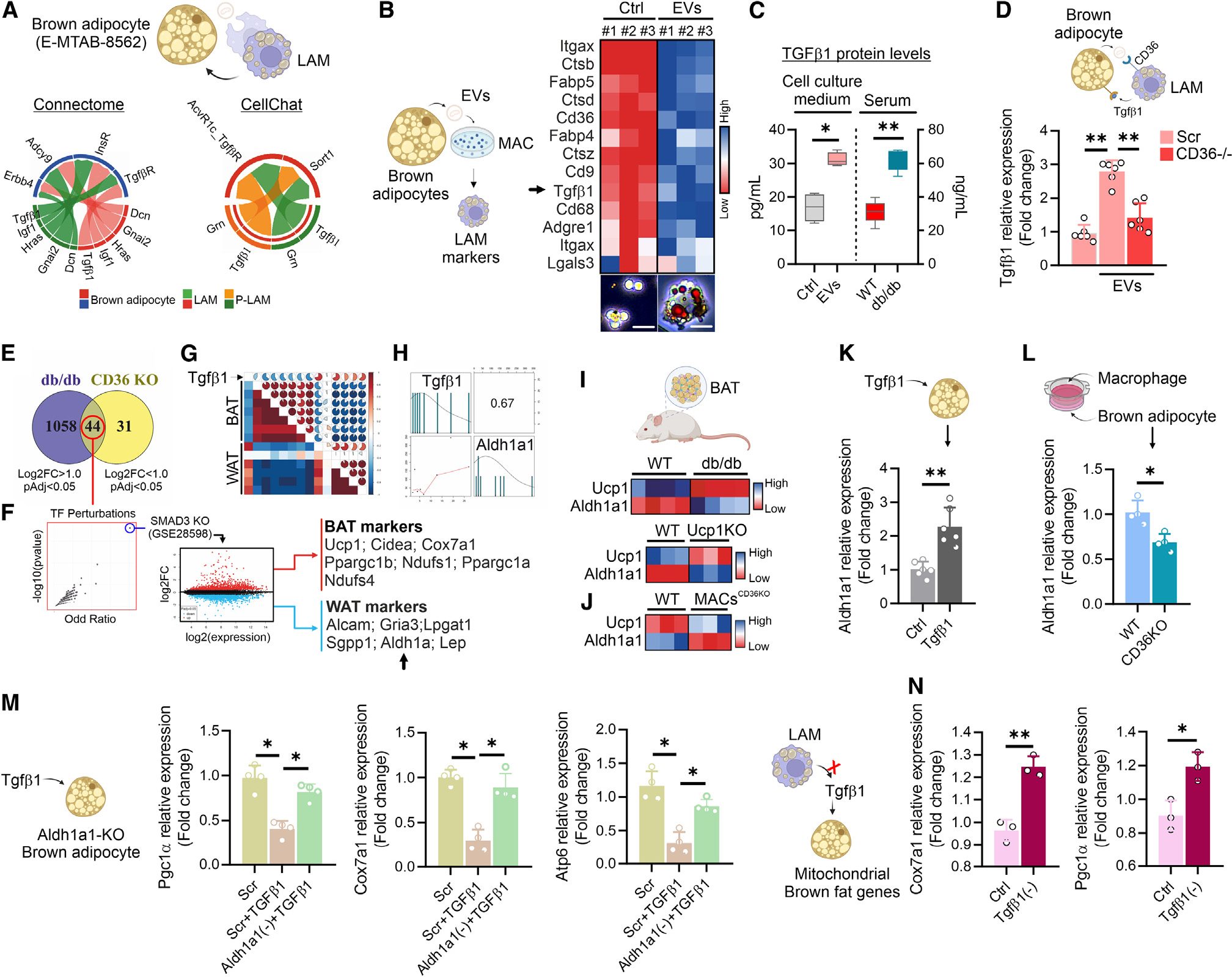
LAM release Tgfβ1 lowering brown-adipocyte identity through Aldh1a1 pathway. (A) Ligand-receptor communication analysis by Connectome and CellChat.^50,51^ (B) LAM-related genes (upper) and representative image of oil red O (lower) in RAW264.7 (MAC) treated with EVs released by brown adipocytes (E-MTAB-10655). (C) Tgfβ1 protein level in cell culture of RAW264.7 macrophages treated with EVs released by brown adipocytes or serum of WT and 8-week-old db/db mice (*n* = 6 mice/group). Data were reported as mean ± SD. Student’s t test *p < 0.05, **p < 0.01. (D) Tgfβ1 mRNA expression in EVs-treated macrophages downregulating CD36. Data were reported as mean ± SD. ANOVA test **p < 0.01. (E) Venn diagram including the upregulated genes in BAT of db/db mice and downregulated genes in BAT of MAC^CD36KO^ mice. (F) Enrichment analysis for transcription factor perturbation related to 44 overlapping genes and differential gene expression analysis in the WAT of SMAD3KO vs. WT mice. (G) Heatmap of correlation analysis between Tgfβ1 and WAT and BAT-related genes in BAT of db/db mice. (H) Correlation analysis between Tgfβ1 and Aldh1a1 in BAT of db/db mice. (I and J) Heatmap reporting gene expression levels in BAT of db/db, Ucp1 KO (I), and MACs^CD36KO^ mice (J) (*n* = 3/4 mice/group). (K) Aldh1a1 mRNA expression in primary brown adipocytes treated with 10 μM Tgfβ1 for 16 h. Data were reported as mean ± SD. Student’s t test **p < 0.01. (L) Aldh1a1 mRNA expression in T37i brown adipocytes cultured with RAW264.7 cells downregulating CD36. Data were reported as mean ± SD. Student’s t test *p < 0.05. (M) Mitochondrial gene expression in primary adipocytes downregulating Aldh1a1 after treatment with 10 μM Tgfβ1 for 16 h. Data were reported as mean ± SD. ANOVA test *p < 0.05. (N) Mitochondrial gene expression in primary adipocytes co-cultured (24 h in serum free) with EVs-treated RAW264.7 macrophages downregulating Tgfβ1. Data were reported as mean ± SD. Student’s t test *p < 0.05, **p < 0.01.

To investigate the mechanism through which LAMs promote molecular reorganization in BAT of db/db mice, we interrogated our scRNA-seq data for ligand-receptor pairs using publicly available computational programs.^43,44^ Through this approach, we predicted that LAMs and P-LAMs communicate with mature brown adipocytes by releasing transforming growth factor β1 (Tgfβ1) (Figure 7A). Single-nuclei data analysis of murine AT^6^ corroborated the idea that Tgfβ1 is mainly expressed in adipose-tissue-resident LAMs and P-LAMs (Figure S7A), with higher levels in obese than lean mice (Figure S7B). Of note, macrophages treated with adipose EVs developed a foaming-like phenotype (Figure 7B) and increased the expression level of LAM markers including Tgfβ1 (Figure 7B). The expression of Tgfβ1 paralleled a significant release of Tgfβ1 from macrophages treated with adipose EVs (Figure 7C), which was consistent with the increased TGFβ1 levels detected in the serum of db/db mice (Figure 7C). Tgfβ1induction was restrained when CD36 was downregulated (Figure 7D), supporting the evidence that Tgfβ1 takes part in CD36-mediated extracellular particle clearance by macrophages.^40,45^ Consistent with this, a similar response was observed when BMDMs were treated with oxLDL (Figure S7C). A significant correlation between Tgfβ1 levels and adiposity in rodents and humans was previously observed.^46^ Moreover, exogenous Tgfβ1 treatment was demonstrated to repress BAT mitochondrial genes promoting ECM synthesis and lipogenesis in adipocytes.^46,47^ Macrophage-derived Tgfβ1 mediates myofibroblast phenotype induction in AT,^48^ and Tgfβ1 antibody protects ob/ob and HFD mice from obesity and T2D.^46^ To test if Tgfβ1 drives the loss of BAT identity in db/db mice, we initially developed a Venn diagram integrating the upregulated genes in BAT of db/db mice with downregulated genes in BAT of MACs^CD36KO^ mice. This analysis led to the identification of 44 overlapping genes pertaining to SMAD3 signaling (Figures 7E and 7F). Of note, Tgfβ1 controls the SMAD3-signal transduction pathway.^46^ Accordingly, we found that ablation of *Smad3* leads to a decrease in the expression levels of WAT-related genes and increase in BAT-related genes (Figure 7F). Next, in order to explore the mechanisms through which Tgfβ1 promotes the loss of BAT identity in db/db mice, we generated a correlative approach linking Tgfβ1 with WAT- and BAT-related genes (Figure 7G). By contrast, we observed an inverse correlation between Tgfβ1 and BAT genes (Figure 7H). In contrast, we observed a positive correlation between Tgfβ1 and WAT genes including Aldehyde Dehydrogenase 1 Family Member A1 (Aldh1a1) (Figures 7G and 7H), which was recently demonstrated to participate in the loss of brown-adipocyte identity.^49^ Accordingly, Aldh1a1 expression was increased in BAT of thermogenic deficient db/db and Ucp1 KO mice (Figure 7I), whereas it was decreased in BAT of thermogenically competent MACs^CD36KO^ mice (Figure 7J). These data were confirmed by co-culture experiments that showed reduced Aldh1a1 levels in mature brown adipocytes cultured with CD36-downregulating macrophages (Figure 7K). Next, we asked if Tgfβ1 controls Aldh1a1 levels in adipose cells. Concomitantly with reduced levels of brown-fat-related genes, Tgfβ1 treatment also increased Aldh1a1 levels in brown adipocytes (Figures 7L and S7D), suggesting a role of LAM-derived Tgfβ1 in mediating the loss of brown-adipocyte identity through Aldh1a1 induction. In order to deepen our comprehension of Aldh1a1’s role in suppressing brown-fat genes, we administered Tgfβ1 to brown adipocytes lacking Aldh1a1 (Aldh1a1 KO). Following Tgfβ1 treatment, we observed an increased expression of mitochondrial genes in Aldh1a1 KO cells compared to controls (Figure 7M). Likewise, when macrophages overexpressing Tgfβ1 were co-cultured with brown adipocytes, they effectively restrained brown-fat identity (Figure S7E). To enhance the understanding of whether Tgfβ1 released from LAM exerts a negative impact on brown-fat genes, we downregulated Tgfβ1 in macrophages treated with EVs and subsequently co-cultured them with adipocytes. Notably, macrophages with reduced Tgfβ1 expression were unable to decrease mitochondrial genes in brown adipocytes (Figure 7N). The overall data highlighted the involvement of LAM in promoting BAT identity reshaping through the Tgfβ1-Aldh1a1 pathway.

## DISCUSSION

This is, to our knowledge, the first study unveiling BAT responses at the single-cell level during obesity. Prior works mainly focused on the role of immune cell dynamics in governing thermogenesis of BAT.^52,53^ Although several findings demonstrated a decreased BAT activity in obese human,^7^ the mechanism leading to such loss of function was unexplored. Major contributors to the AT dysfunction during obesity are tissue-resident macrophages.^54–56^ Under both homeostatic and pathological conditions, AT is interspersed with a large range of immune cells, including macrophages, which dramatically increase in total abundance with greater adiposity.^5^ Through scRNA-seq, a massive dynamic of adipose tissue-resident macrophages has been demonstrated in both mouse and human models of obesity.^57^ Specifically, macrophages develop a peculiar transcriptional signature resembling LAMs, which are characterized by high expression of genes related to lipid metabolism and phagocytosis (i.e., *Trem2, Lipa, Lpl, Ctsb, Ctsl, Fabp4, Fabp5,* and *Cd36*).^4–6^ It has been demonstrated that *in vivo* depletion of Trem2^+^ macrophages exacerbate the metabolic abnormalities in obese mice.^5,58^ Recent findings report that Trem2^+^ macrophages participate in heart functions by scavenging dysfunctional mitochondria ejected from cardiomyocytes.^20^ However, few reports have described the macrophage features and functions in BAT during obesity.^22,59^ Through analysis of RNA and surface protein expression, we identified the accumulation of LAMs in BAT of obese mice. While monocytes/macrophages physiologically clear extracellular debris released from metabolically stressed brown adipocytes,^16^ they develop foam cell-like characteristics upon removing substantial quantities of mitochondria- and lipid-filled EVs, especially in the context of obesity or T2D. Although BAT shows a strong capacity to maintain metabolic flexibility through the breakdown of circulating glucose and lipoproteins,^13,14^ chronic and excessive nutrient intake induces the expansion of BAT, which manifests a WAT-like phenotype, reminiscent of the metabolic changes observed in BAT transitioning from cold temperatures to thermoneutral conditions.^60,61^ We hypothesize that this expansion of BAT serves as an adaptive mechanism to alleviate the metabolic stresses linked to conditions such as obesity and T2D. In such metabolic circumstances, a malfunction in lysosomal activity may foster the accumulation of oxidatively damaged mitochondria and lipids,^62^ which are swiftly released into the extracellular milieu as a mechanism to uphold cellular quality control.^63^ Consistent with this, selective lysosomal inhibition increased secretion of mitochondria in large EVs, which are captured by macrophages without activating inflammation.^19^ Mature adipocytes with a deletion of autophagy genes (Atg3 or Atg16L1) accumulate dysfunctional mitochondria and increase the extracellular release of lipid peroxides.^17^ Similarly, adipocytes with defective lysosomal clearance increase the release of mitochondria and lipids via EVs.^16,18^ While lack of Trem2^+^ macrophages is detrimental in WAT, causing hypertrophy and glucose intolerance, CD36^+^ lack of macrophages in BAT improves brown-adipocyte function, promoting the expression of oxidative genes. However, whether Trem2 and CD36 mediate a similar function or whether the different responses were related to different ATs remains an open question that needs further investigation. CD36 downregulation in macrophages was efficient to enhance the brown-fat gene expression, highlighting a role of this subtype of LAMs in mediating white-like features of BAT. We suppose that Tgfβ1 released from CD36^+^ LAM participates in reducing the expression of brown-fat genes. CD36^+^-downregulating macrophages limited Tgfβ1 induction following foaming stimuli such as treatment with oxLDL or adipocyte EVs. It has been reported that Tgfβ1 promotes ECM remodeling, intracellular lipogenesis, and de-differentiation of mature adipocytes.^47,64^ More recent data also demonstrated that Tgfβ1 induces whitening phenotype in beige adipocytes.^65^ A positive correlation between Tgfβ1 levels and adiposity in rodents and humans was observed. Our data indicate that macrophage-derived Tgfβ1 promotes Aldh1a1 in brown adipocytes, which is consistent with recent findings demonstrating that Aldh1a1 is exclusively expressed in mature adipocytes and that its loss has been associated with increased brown-adipocyte function.^49^ In conclusion, our findings underscore the role of LAMs in the expansion of BAT, which contributes to the transition of BAT from its functional state to a WAT-like phenotype during obesity.

### Limitations of the study

There are several notable limitations to our study. Firstly, we lack a detailed understanding of the mechanism through which EVs are produced and released from fat cells. Additionally, there is a need for a comprehensive characterization of these EVs. Although we have extensively examined the molecular responses of brown adipocytes to metabolic overload, both *in vitro* and *in vivo* bioenergetic profiling remains unexplored. Furthermore, while we have identified Tgfβ1 as a critical mediator in the loss of brown-fat identity by LAMs, there is a lack of *in vivo* models demonstrating whether macrophage-derived Tgfβ1 specifically controls the identity of brown adipocytes. Considering that LAMs may release various other factors, such as metabolites and EVs, we cannot disregard the possibility that these molecules also contribute to the loss of BAT identity during obesity. Additionally, in our study, the immune pathway responsible for the accumulation of LAMs in BAT of obese mice has not been experimentally validated. Lastly, a crucial aspect not addressed in our study is the analysis of responses from non-adipocyte macrophages, such as Kupffer cells, which are known to play a significant role in the metabolic regulation of obese animals.

## STAR★METHODS

### RESOURCE AVAILABILITY

#### Lead contact

Further information and requests for resources and reagents should be directed to and will be fulfilled by the lead contact: Daniele Lettieri-Barbato (daniele.lettieri.barbato@uniroma2.it).

#### Materials availability

This study did not generate unique reagents.

#### Data and code availability

- All raw data that support the findings of this study are available from the lead contact upon reasonable request. scRNA-seq and bulk RNA-seq datasets produced in this study are available from gene expression omnibus (GEO) with accession numbers GSE232278 and GSE177635. The newly generated proteomic data have been deposited into the ProteomeXchange database with the following accession number ProteomeXchange: PXD042126.
- This paper does not report original code.

### EXPERIMENTAL MODEL AND STUDY PARTICIPANT DETAILS

#### Animal experiments

##### Obese mouse models, MitoFAT, Ucp1^Δ/Δ^ and MACs^CD36KO^

Wild type C57BL6/J male mice were obtained from Charles River Laboratories. Unless otherwise indicated, all experiments were performed in male mice kept on an inverted 12-h:12-h dark:light cycle. For consistency, all mice used in this study were males. All mice were provided with normal diet and water *ad libitum* and housed under a strict 12-h light-dark cycle. At age 10 weeks, for some mice the normal diet was replaced with a high-fat diet (HFD; irradiated Rodent Diet with 60 kcal% fat, D12492i Research Diets Inc., New Brunswick, NJ) for 10 weeks. Type 2 diabetic B6.BKS(D)-Leprdb/J (db/db) mice were purchased from Jackson laboratories and sacrificed at 8 and 16 weeks at 8 and 16 weeks of age. Biochemical parameters and tissue harvesting were conducted 16 h after fasting. Experiments were approved by University of Rome Tor Vergata Animal Care Italian Ministry of Health Committee (protocol 256/2022-PR). mtDendra2 and Adipoq-Cre^+/−^ mice were obtained from Jackson Laboratories and were on a C57BL6/J background. mtD2^Flox/Flox^ mice were crossed with AdipoqCre^/+^ mice to generate mtDendra2Flox^/+^ AdipoqCre^/+^ (MitoFAT) and mtDendra2Flox/+ (control) mice. Adult mice fed with normal diet was replaced with an HFD for 10 weeks. All experiments were carried out under the guidelines of the Institutional Animal Care and Use Committee (IACUC) at Washington University in St. Louis and were performed under IACUC-approved protocols. Ucp1^Δ/Δ^ were generated by crossing Pnpla2^fl/fl^ [B6N.129S-Pnpla2tm1Eek/J] and Ucp1^Cre^ [B6.FVBTg(Ucp1-cre)1Evdr/J] mice (all mice were obtained from Jackson Laboratories and were on a C57BL6/J background; Ucp1^cre^ mice were kindly provided by Dr. Jean-François Tanti). Animal protocols were approved by the Institutional Animal Care and Use Committee of the French Ministry of Higher Education and Research and the Mediterranean Center of Molecular Medicine (INSERM U1065) and were undertaken in accordance with the European Guidelines for Care and Use of Experimental Animals. Myeloid-specific deficiency (CD36^flox/flox^ × Csf1r^Cre^) mouse models (MACs^CD36KO^) were kindly provided by Susan M. Kaech (NOMIS Center for Immunobiology and Microbial Pathogenesis, Salk Institute for Biological Studies, La Jolla, CA 92037, USA) and sacrificed at 10 weeks of age. Mice were sacrificed by cervical dislocation and the explanted BAT was directly used for processing.

#### Cell culture

##### Murine T37i and primary brown adipocytes

T37i murine preadipocytes were kindly provided by Professor Marc Lombes (INSERM U1185, Paris, France) and cultured in Dulbecco’s modified Eagle’s medium Nutrient Mixture F-12 (DMEM/12), 10% heat-inactivated fetal bovine serum (FBS), and 100 U/mL penicillin and 10 mg/mL streptomycin (1% P/S) (Life Technologies, Carlsbad CA, USA). For differentiation, cells were maintained at complete confluency. After 2 days, 2 nM triiodothyronine (T3, Sigma-Aldrich), 1 mM rosiglitazone, and 1 mg/mL insulin (Sigma-Aldrich) were added to fresh medium every 2 days up to 8 days.

Primary brown adipocytes were isolated and differentiated as described by.^71^ Interscapular BAT from 4-week-old male mice were excised and submerged in washing buffer (HBSS with 3.5% w/v BSA, 1% P/S, 40 mg/mL gentamicin, and 500 ng/mL amphotericin B). After mincing, BAT was digested in a collagenase solution (HBSS with 3.5% w/v BSA, 1% P/S, 0.1% w/v collagenase type 1, 0.1% w/v collagenase type 2, 40 mg/mL gentamicin, and 500 ng/mL amphotericin B) by incubating at 37°C with shaking at 150 rpm for 1 h. BAT homogenates were then centrifuged twice at 250 3 g × 5 min at RT, and the resulting pellet was resuspended in 3 mL of red blood cell lysis buffer (ThermoFisher Scientific; Rockford, IL, USA) for 4 min. Successively, 10 mL of washing buffer was added, and the cell suspension was filtered through a 40-mm cell strainer. The cell suspension was centrifuged at 500 g × 5 min at RT, and the pellet was resuspended in growth medium (DMEM/12 with 10% FBS, 1% P/S, 40 mg/mL gentamicin, and 500 ng/mL amphotericin B) until 60% confluency was attained and then plated. Twenty-four hours after attaining 100% confluence, brown adipocytes were differentiated by treatment with an induction medium (DMEM/12 with 10% FBS, 1% P/S, 40 μg/mL gentamicin, 500 ng/mL amphotericin B, 850 nM insulin, 1 nM T3, 1 mM rosiglitazone, 1 mM dexamethasone, 500 mM 3-isobutyl-1-methylxanthine (IBMX), and 125 mM indomethacin) for 48 h. On day 2, the induction medium was removed, and a differentiation medium (DMEM/12 with 10% FBS, 1% P/S, 40 μg/mL gentamicin, 500 ng/mL amphotericin B, 850 nM insulin, 1 nM T3, 1 and mM rosiglitazone) was added and maintained for 6 days. Prior to treatments or collection of extracellular vesicles (EVs), mature brown adipocytes (day 8) were treated with 400 μM BSA-conjugated palmitic acid (PA) or 1 μM trifluoromethoxy carbonylcyanide phenylhydrazone (FCCP) or 1 μM antimycin A (AA) or 25 mM chloroquine (CQ). Primary brown adipocytes cells were transfected with 200 pmol of small interfering RNA (Aldh1a1 or scramble sequence) using Lipofectamine 2000 transfection reagent, according to manufacturer’s instructions (ThermoFisher).

#### Murine RAW 264.7 and bone marrow derived macrophages (BMDM)

Murine RAW 264.7 macrophages were cultured in DMEM supplemented with 10% FBS and 1% P/S (Life Technologies) at 37°C in a humidified incubator containing 5% CO2. Twenty-four hours after plating, RAW264.7 cells were treated with EVs collected from brown adipocytes, maintaining a cell ratio of 1:5. A similar ratio was maintained by co-culturing RAW264.7 cells with T37i brown adipocytes. Co-culture system was conducted with 400 μM PA in the cell culture medium. Prior to treatments RAW264.7 cells were treated with 25 mM chloroquine (CQ). RAW264.7 cells were transfected with 200 pmol of small interfering RNA (CD36, Tgfβ1 or scramble sequences) or 2 μg/mL of expression plasmid (Tgfβ1 or empty vector) using Lipofectamine 2000 transfection reagent, according to manufacturer’s instructions (ThermoFisher).

Bone marrow (BM) was extracted from the limbs of 8-week-old male mice by perfusion with PBS and 1% P/S. Bone marrow-derived macrophages were plated at a density of 3 × 10^5^ cells/mL in alpha-MEM supplemented with 10% FBS, 1% P/S, and 1% GlutaMAX in a humidified incubator containing 5% CO2 at 37°C. Macrophage differentiation was induced by adding M-CSF (20 ng of cells/mL) in the culture medium for 8 days. Following adhesion, unattached cells were removed, and BMDM were treated with brown adipocytes EVs. At the end of the treatments, macrophages were harvested, and proteins or RNA were extracted as described below.

### METHOD DETAILS

#### Extracellular vesicle isolation

EVs from BAT and brown adipocytes were isolated as previously described.^16^ Firstly, before isolating EVs, the white adipose tissue surrounding the BAT was adequately removed. Subsequently, BAT or brown adipocytes were cultured in serum-free DMEM. Culture media were collected and centrifuged at 600 g × 10 min at 4°C to remove dead cells. Successively, the supernatant was centrifuged at 100,000 g for 16 h at 4C (Rotor SW28 or SW40, according to sample volume; Beckman Coulter, CA, USA) EVs pellets were washed once with PBS or radioimmunoprecipitation assay (RIPA) buffer for treatments or immunoblotting, respectively. Protein determination was performed using the Lowry method.

#### scRNAseq, cell clustering and cell-type annotation, cell–cell ligand–receptor interaction analysis, MacSpectrum, AtheroSpectrum, velocyto and scVelo

Single-cell suspensions were obtained from the stromal vascular fraction (SVF), which was isolated from BAT following the removal of the surrounding white adipose tissue capsule. Single-cell suspensions were prepared for scRNA-seq immediately after cell sorting using a Chromium Single-Cell Reagent Kit according to the manufacturer’s protocol (10x Genomics). Following capture and lysis, cDNA was synthesized for each droplet of captured cells and amplified (12 cycles). The amplified cDNA from each channel of the Chromium system was used to construct an Illumina sequencing library and was sequenced on a NovaSeq 6000 with 150-cycle sequencing (asymmetric reads per 10x Genomics). Single-cell libraries were prepared using 10x Genomics Chromium 3′ reagent kit according to manufacturer’s protocol. Libraries were sequenced using the NovaSeq 6000 (Illumina) to a depth of approximately 300 million reads per library with 2 × 50 read length. Raw reads were aligned to the *Mus musculus* (mm10) and cells were called using CellRanger count (v.7.1.0). Individual samples were aggregated to generate the merged digital expression matrix using CellRanger (v7.0.1). The barcodes, features, matrix files generated by CellRanger software were used as input into the R program Seurat (v4.3.0).^30^ Low quality cells were filtered out retaining only cells with features number greater than 250, gene counts greater than 500 and mitochondrial percentage less than 15. Outliers were also removed (features >6000 and counts >30000) keeping genes expressed by more than 3 cells. The same number of cells (5500 from each sample) was randomly subsampled from the filtered barcodes. Expression levels were normalized by logarithmic reduction. Next, the most variable genes were identified (2000 features selected). The different samples (WT, db/db 8weeks, db/db 16weeks, HFD) were integrated in a single object using the Canonical Correlation Analysis. The gene expressions were scaled among all cells. The dimensionality of the object was studied with the Principal Component Analysis, then the first 15 principal components were used to generate the UMAP reduction. Clusterization was obtained setting a 0.5 resolution. The differentially expressed genes of each cluster were identified using the Wilcoxon rank-sum test. Each cluster was manually labeled, annotation confirmed by SingleR using CellDex libraries (v1.10.0).^69^ Macrophages polarization and foaming were conducted with MacSpectrum (v0.1.0)^35^ and AtheroSpectrum (v1.0.1)^34^ tools, respectively. Macrophage subclustering was conducted with ClusterProfiler (v4.4.4)^70^ and the enrichment analysis was performed using Gene Ontology database. Trajectories were calculated and plotted by Monocle3 (v1.3.1).^31^ Cell–cell ligand–receptor interaction analysis was investigated by CellChat (v1.6.1)^50^ and Connectome (v1.0.1)^72^ packages. GGPlot2 (v3.4.0) and SCpubR (v1.0.4) were used to generate graphs. Velocity analyses were conducted using two distinct tools: Velocyto^32^ and ScVelo.^73^ The calculation of velocities employed velocyto (v0.17.17), with BAM files provided as input to generate unspliced and spliced abundance counts in loom format.

For the inference of gene-specific RNA velocities encompassing transcription, splicing, and degradation rates, we employed scVelo (v0.2.5) to explore cell trajectories. The loom files underwent preprocessing through scvelo’s dedicated functions. Velocity calculations were executed using a likelihood-based dynamical model, and subsequently, the velocity vectors were projected onto the UMAP projection of the cells.

#### Bulk RNA-sequencing and functional enrichment analysis

Total RNAs from cells and tissues were extracted using Direct-zolTM RNA MiniPrep (ZYMO RESEARCH) according to the manufacturer’s instructions. Total RNA was quantified using the Qubit 4.0 fluorimetric Assay (Thermo Fisher Scientific). Libraries were prepared from 50 ng of total RNA using the NEGEDIA Digital mRNA-seq research grade sequencing service (Next Generation Diagnostic srl), which included library preparation, quality assessment and sequencing on a NovaSeq 6000 sequencing system using a singleend, 75 cycle strategy (Illumina Inc.). The raw data were analyzed by Next Generation Diagnostic srl proprietary NEGEDIA Digital mRNA-seq pipeline (v2.0) which involves a cleaning step by quality filtering and trimming with bbduk, alignment to the reference genome (mm10) using STAR 2.6.0a and counting by gene with HTseq-counts 0.9.1. The raw expression data were normalized and analyzed by Rosalind HyperScale architecture 2^74^(OnRamp BioInformatics, Inc.). Differentially expressed genes were calculated by DESeq2 package (v1.40.1).^74^ Gene correlation analysis was conducted using CorrPlot (v0.92) and Psych tools (v2.3.3). The bidirectional hierarchical clustering heatmap was generated using FunRich software (version 3.1.3).^75^ Functional enrichment analysis was performed by the Enrichr analysis tool^76^ and graphic displaying was performed by https://www.bioinformatics.com.cn/en, a free online platform for data analysis and visualization. Additional enrichment analysis was performed by ClusterProfiler (v4.4.4)^70^ using Gene Ontology database. Thermogenic potential of bulk RNA samples was tested with ProFAT online tool.^28^

#### qPCR gene expression analysis

Total adipose RNA was isolated using TRIzol reagent and purified using the RNeasy mini kit protocol according to the manufacturer’s instructions. Then, 1 μg of RNA was treated with genomic DNase and retro-transcripted using PrimeScript RT Reagent Kit (Takara Bio Inc., Japan). qPCR was performed in triplicate on 50 ng of cDNA using validated qPCR primers (BLAST), Applied Biosystems Power SYBR Green Master Mix, and the QuantStudio3 Real-Time PCR System (Thermo Fisher, Waltam, MA, USA), as described by Turchi et al. (2020). The primers used for qPCR are included in the Table S4.

The reaction was performed according to the manufacturer’s protocol using QuantStudio 3 Real-Time PCR System. Data were analyzed following the 2^−ΔΔ^Ct method.

#### Untargeted proteomics

Cells or EVs were harvested as indicated in the text and directly lysed in ice-cold RIPA buffer. Samples preparation was done using the in StageTip (iST) method.^77^ Samples were separated by HPLC in a single run (without pre-fractionations) and analyzed by LC-MS/MS. Instruments for LC-MS/MS analysis consisted of a NanoLC 1200 coupled via a nano-electrospray ionization source to the quadrupole-based Q Exactive HF benchtop mass spectrometer. Peptide separation was carried out according to their hydrophobicity on a home-made column, 75 mm ID, 8 Um tip, 400 mm bed packed with Reprosil-PUR, C18-AQ, 1.9 mm particle size, 120 Angstrom pore size (New Objective, Inc., cat. PF7508–250H363), using a binary buffer system consisting of solution A: 0.1% formic acid and B: 80% acetonitrile, 0.1% formic acid. Total flow rate: 300 nL/min. LC linear gradient: after sample loading, run start at 5% buffer B for 5 min, followed by a series of linear gradients, from 5% to 30% B in 90 min, then a 10 min step to reach 50% and a 5 min step to reach 95%. This last step was maintained for 10 min. MS spectra were acquired using 3E6 as an AGC target, a maximal injection time of 20 ms and a 120,000 resolution at 200 m/z. The mass spectrometer operated in a data dependent Top20 mode with subsequent acquisition of higher-energy collisional dissociation (HCD) fragmentation MS/MS spectra of the top 20 most intense peaks. Resolution for MS/MS spectra was set to 15,000 at 200 m/z, AGC target to 1E5, max injection time to 20 ms and the isolation window to 1.6 Th. The intensity threshold was set at 2.0E4 and Dynamic exclusion at 30 s. All acquired raw files were processed using MaxQuant (1.6.2.10) and the implemented Andromeda search engine. For protein assignment, spectra were correlated with the Human (v. 2021) including a list of common contaminants. Searches were performed with tryptic specifications and default settings for mass tolerances for MS and MS/MS spectra. The other parameters were set as follow: fixed modifications: carbamidomethyl (C); variable modifications: oxidation, acetyl (N-term); digestion: trypsin, Lys-C; min. peptide length = 7; max. peptide mass = 470 Da; false discovery rate for proteins and peptide-spectrum = 1%.

For further analysis, the Perseus software (1.6.2.3)^78^ was used and first filtered for contaminants and reverse entries as well as proteins that were only identified by a modified peptide [First filter]. The LFQ Ratios were logarithmized, grouped and filtered for min.valid number (min. 3 in at least one group) [Second filter].

Missing values have been replaced by random numbers that are drawn from a normal distribution. Two - sample T test analysis was performed using FDR = 0.01. Proteins with difference Log_2_ Difference≥ ± 1 and q value < 0.01 were considered significantly enriched. Proteomics data were integrated with transcriptomics data by 2D density plot in R and ggplot2.

#### BAT immune cell isolation

Immune cells were isolated from BAT as previously described.^16^ Briefly, before tissue processing, the white adipose tissue surrounding BAT was thoroughly removed. Subsequently, BAT was finely minced with scissors and then incubated in a high-glucose solution DMEM containing 0.1% collagenase II (Sigma-Aldrich) at 37°C in an orbital shaker at ~150 rpm for 1 h. The cell suspension was filtered through a 100-μm nylon mesh and cells were collected in a 50-mL conical tube and centrifuged at 500 × g for 5 min at 4°C. The supernatants and floating adipocytes were aspirated, and the SVF pellet was resuspended in 1 mL of ACK RBC lysis buffer (Gibco) and incubated at RT for 2 min. The RBC lysis reaction was quenched by adding 10 mL of cold wash media (high-glucose DMEM containing 5% heat-inactivated FBS, L-glutamine, and penicillin/streptomycin). The cells were centrifuged at 500 × g for 5 min at 4°C and the supernatants were aspirated. Cells were resuspended in 200 mL of wash media for subsequent staining on a polystyrene 96-well round bottom tissue-culture treated plate. The cells were centrifuged at 500 × g for 5 min at 4°C, and the cell pellet was washed in PBS once and centrifuged again as described above. SVF pellet was then split and either resuspended in a mixture of 9:1 fetal bovine serum:dimethylsulfoxide solution and frozen at −80°C for scRNAseq or stained using fluorescently labeled antibodies, and then analyzed via flow cytometry.

#### High dimensional flow cytometry and cell sorting

Cells were incubated with FcBlock (1:100, BD Pharmigen in FACS buffer: 200 mL; PBS containing 2.5% heat inactivated FBS and 2.5 mM EDTA) on ice for 10 min, and then an equal volume of a mix cocktail containing >20 markers was added and mixed, as reported.^16,79^ Briefly, after 30 min of staining on ice (protected from light), the cells were washed 2–3 times in cold PBS and acquired by flow cytometers and analyzed by FlowJo software version 10. Macrophages were identified as CD45+/CD11b+/F4.80+/CD64+CD11c−Ly6C− cells and inside this gate, the expression of several markers defining cell subsets such as inflammatory status, phagocytosis and lipid-handling ability was evaluated by staining with anti-CD36, anti-CD9. Monocytes were identified with several pan and subset markers, such as anti-Ly6C, anti-CX3CR1, anti-CCR2, anti-CD115, anti-CD62L and MHC-II. The list of antibodies is shown in Table S5. Samples were acquired on a 21-color Cytoflex (Beckman Coulter), BD X20 flow cytometer or Cytek Aurora spectral flow cytometer and for each analysis, at least 0.5 × 10^6^ live cells were acquired by gating on viable cells using either Zombie UV (1:1600) or Zombie NIR (1:1000).

After adipose tissue dissociation into single cell suspension, cells were stained with BODIPY 493/503, LysoTracker red, anti-CD45 BV510, anti- CD11b PE-Cy7, anti-CD64 BV711, anti-CD36 PerCP5.5 and anti-CD9 PE. Leukocytes within adipose tissue were gated on CD45 and macrophages were identified as CD11b+CD64^+^. CD36^+^CD9^+^ LAM and CD36^−^CD9^−^non-LAM were sorted by FACS Astrios Cell Sorter (Beckman Coulter) and used for subsequent molecular analysis.

#### Mitochondrial labelling

To stain mitochondria, mature brown adipocytes were loaded with MitoTracker Green and Red (ThermoFisher Scientific) for 1 h. After *in vitro* treatments, EVs were collected and to eliminate unincorporated dye from EVs, exosome spin columns were used to remove low MW (≤3,000 Da) admixtures (4484449, ThermoFisher Scientific). Alternatively, brown adipocytes were transfected with 2μg/well of plasmid carrying mitochondrial dsRed (mitoDsRed) using Lipofectamine 2000 transfection reagent, according to manufacturer’s instructions (ThermoFisher). Flow cytometry analyses were performed using Amnis CellStream (Luminex, USA) and analyzed using FlowJo software version 4.14.

#### Histochemical analysis

Formalin-fixed paraffin-embedded BAT explants were cut into 3 μm sections and stained with hematoxylin and eosin (H&E) prior to microscope analysis. After antigen retrieval with citrate buffer (pH 6.0), sections were incubated at room temperature with the following primary antibody anti-F4/80. Negative control was obtained by omitting primary antibodies. Immunohistochemical reactions were visualized by DAB as the chromogen from MACH 1 Universal HRP-Polymer Detection (Biocare Medical, Concord, MA, USA).

#### Immunoblotting

Tissues or cells were lysed in RIPA buffer (50 mM Tris-HCl, pH 8.0, 150 mM NaCl, 12 mM deoxycholic acid, 0.5% Nonidet P-40, and protease and phosphatase inhibitors). Then, 10–20 μg of proteins were loaded on sodium dodecyl sulfate–polyacrylamide gel electrophoresis (SDS-PAGE) and subjected to immunoblotting. Nitrocellulose membranes were incubated with primary antibodies at a dilution of 1:1000. The membranes were then incubated with the appropriate horseradish peroxidase-conjugated secondary antibodies. Immunoreactive bands were detected using a FluorChem FC3 System (Protein-Simple, San Jose, CA, USA) after incubating the membranes with ECL Select Western Blotting Detection Reagent (GE Healthcare, Pittsburgh, PA, USA). Densitometric analyses of the immunoreactive bands were performed using FluorChem FC3 Analysis software. Representative immunoblots of at least *n* = 3 independent experiments or mice/group are shown.

### QUANTIFICATION AND STATISTICAL ANALYSIS

Data were expressed as the mean ± SD unless otherwise stated. The exact numbers of replicates are given in each figure legend. A two-tailed unpaired Student’s t-test was performed to assess the statistical significance between two groups. Analysis of variance (ANOVA) followed by Dunnett’s (comparisons relative to controls) or Tukey’s (multiple comparisons among groups) post hoc tests was used to compare three or more groups. Venn diagrams were constructed using Draw Venn Diagram or Venny 2.1.0 software. Statistical analyses were performed using GraphPad Prism 9 (GraphPad Software Inc., San Diego, CA, USA). In all cases, a *p* value or FDR less than 0.05 was set as the minimum significance threshold.

## Supplementary Material

1

2

3

4

## Figures and Tables

**KEY RESOURCES TABLE:** 

REAGENT or RESOURCE	SOURCE	IDENTIFIER

Antibodies		

4 Hydroxynonenal antibody, Abcam	Abcam	Cat# ab46545, RRID: AB_722490
Anti-CD36 antibody	Abcam	Cat# ab124515, RRID:AB_2924667
Anti-F480	Abcam	Cat# ab300421, RRID: AB_2936298
Flow cytometry antibodies list	Table S5	N/A
P62	Santa Cruz	Cat# sc-10117, RRID: AB_2195900
LC3	Sigma	Cat# L7543, RRID: AB_796155
ATGL	Cell Signaling	Cat# 2439, RRID: AB_2167953
PLIN	Santa Cruz Biotechnology	Cat# sc-67164, RRID: AB_2252681
HSL	Cell Signaling	Cat# 4107S, RIID: AB_2296900
Pyruvate Dehydrogenase E1 beta subunit antibody	GeneTex	Cat# GTX119625, RRID: AB_11163683
Beta Tubulin antibody	Proteintech	Cat# 10094–1-AP, RRID: AB_2210695
Beta Actin Polyclonal antibody	Proteintech	Cat# 20536–1-AP, RRID: AB_10700003
Anti-CD63 Antibody, System Biosciences	System Biosciences	Cat# EXOAB-CD63A-1, RRID: AB_2561274
Vinculin Monoclonal Antibody (VLN01)	Thermo Fisher Scientific	Cat# MA5–11690, RRID: AB_10976821

Bacterial and virus strains		

mitoDs-RED	Addgene	Plasmid #55838 RRID: AB_Addgene_55838

Chemicals, peptides, and recombinant proteins		

Chloroquine diphosphate salt	Sigma Aldrich	#C6628
DNase I	Sigma Aldrich	Cat# 10104159001
3,3',5-Triiodo-L-thyronine sodium salt powder, BioReagent, suitable for cell culture	Sigma Aldrich	Cat# T6397
Rosiglitazone, ≥98% (HPLC)	Sigma Aldrich	Cat# R2408
Insulin solution human	Sigma Aldrich	Cat# I9278
Collagenase, Type I, powder	Thermo Fisher Scientific	Cat# 17100017
Collagenase, Type II, powder	Thermo Fisher Scientific	Cat# 17101015
eBioscience^™^ 1X RBC Lysis Buffer	Thermo Fisher Scientific	Cat# 00–4333-57
3-Isobutyl-1-methylxanthine, BioUltra, ≥99%	Sigma Aldrich	Cat# I7018
Indomethacin	Sigma Aldrich	Cat# I7378
Cl 316,243 hydrate, ≥98% (HPLC), powder	Sigma Aldrich	Cat# C5976
Carbonyl cyanide 4-(trifluoromethoxy)phenylhydrazone, ≥98% (TLC), powder	Sigma Aldrich	Cat# C2920
N-Acetyl-L-cysteine, cell culture tested, BioReagent, CAS 616–91–1	Sigma Aldrich	Cat# A9165
Doxycycline hyclate, ≥98% (HPLC)	Sigma Aldrich	Cat# D9891
Puromycin	Invivogen	Cat# ant-pr-1
Antimycin a from Streptomyces sp.	Sigma Aldrich	Cat# A8674
Lipopolysaccharides from Escherichia coli O111:B4, gamma-irradiated, BioXtra	Sigma Aldrich	Cat# L4391
LysoTracker red	Thermo Fisher Scientific	Cat# L12492
Mito Tracker Green	Thermo Fisher Scientific	Cat# M7514
MitoTracker^™^ Red CMXRos	Thermo Fisher Scientific	Cat# M7512
BODIPY 493/503	Thermo Fisher Scientific	Cat# D3922
Recombinant Mouse MCSF (Animal-Free)	Cell Guidance Systems	Cat# GMF8AF

Critical commercial assays		

Lipofectamine^™^ 2000 Transfection Reagent	Thermo Fisher Scientific	Cat# 11668019
OctoMACS^™^ Starting Kit	Miltenyi Biotec	Cat# 130–042-108
QuadroMACS^™^ Starting Kit (LS)	Miltenyi Biotec	Cat# 130–091–051
MS Columns	Miltenyi Biotec	Cat# 130–042–201
LS Columns	Miltenyi Biotec	Cat# 130–042–401
Pre-Separation Filters (30 mm)	Miltenyi Biotec	Cat# 130–041–407
RNeasy Lipid Tissue Mini Kit	Qiagen	Cat# 74804
Maxpar 10X Barcode Perm Buffer	Fluidigm	Cat# 201057
Cell-ID^™^ 20-Plex Pd Barcoding Kit	Fluidigm	Cat# 201060
Maxpar^®^ Cell Staining Buffer	Fluidigm	Cat# 201068
Cell-ID^™^ Intercalator-Ir	Fluidigm	Cat# 201192A
EQ Four Element Calibration Beads	Fluidigm	Cat# 201078

Deposited data		

Single-Cell/RNAseq data (Gene Expression Omnibus)	https://www.ncbi.nlm.nih.gov/geo/	GSE232278
PRoteomics IDEntification database (PRIDE)	https://www.ebi.ac.uk/pride/	PXD042126

Experimental models: Cell lines		

T37i murine preadipocytes	Professor Marc Lombes (INSERM U1185, Paris, France)	N/A
RAW 264.7 cell line	ATCC	Cat# TIB-71, RRID:AB_CVCL_0493

Experimental models: Organisms/strains		

C57BL/6J	The Jackson Laboratory	#000664, RRID: AB_IMS_JAX:000664
Ucp1^Δ/Δ^	Gallerand et al.^22^	N/A
MACs^CD36KO^	Salk Institute for Biological Studies, La Jolla, CA, USA	N/A
db/db	The Jackson Laboratory	#000642, RIID: AB_IMSR_JAX:000642
MitoFat	Brestoff et al.^23^	N/A

Oligonucleotides		

RT-qPCR Primer List	Table S4	N/A
Tgfβ1 siRNA (mouse)	Origene	Cat# SR413084
Tgfβ1 expression plasmid (mouse)	Origene	Cat# MR227339L1
Cd36 siRNA (mouse)	Origene	Cat# SR426922

Software and algorithms		

Desktop Wave Software	Agilent Technologies	N/A
FunRich version 3.1.3	http://www.funrich.org	N/A
EnrichR	https://amp.pharm.mssm.edu/Enrichr	N/A

Olympus ITEM software	OLYMPUS	N/A
Fiji ImageJ	https://imagej.net/software/fiji/downloads	N/A
FastQC version 0.11.5	https://www.bioinformatics.babraham.ac.uk/projects/fastqc	N/A
Trimmomatic version 0.36	Bolger et al.^66^	N/A
HISAT2 version 2.1.0	Kim et al.^67^	N/A
StringTie version 1.3.4d	Pertea et al.^68^	N/A
AnnotationDbi R library	http://bioconductor.org	N/A
MultiQuant^™^ software version 3.0.2	https://download.sciex.com/MultiQuant_302_Software_Release_Notes.pdf	N/A
Gene Expression Omnibus database	https://www.ncbi.nlm.nih.gov	N/A
Single Cell Portal	https://singlecell.broadinstitute.org/single_cell	N/A
CellRanger version 7.0.1	https://support.10xgenomics.com/single-cell-gene-expression/software/overview/welcome	N/A
R software for statistical computing version 4.3.0	https://www.r-project.org/	N/A
R Studio IDE version 2023.03.1 Build 446	https://posit.co/products/open-source/rstudio/	N/A
Seurat version 4.3.0	Satija et al.^30^	N/A
SingleR	Aran et al.^69^	N/A
CellDex Library version 1.10.0	http://bioconductor.org/packages/release/data/experiment/html/celldex.html	N/A
MacSpectrum version 0.1.0	Li et al.^35^	N/A
AtheroSpectrum version 1.0.1	Li et al.^34^	N/A
ClusterProfiler version 4.4.4	Yu et al.^70^	N/A
Gene Ontology DataBase	http://geneontology.org/	N/A
Monocle3 version 1.3.1	Trapnell et al.^31^	N/A
CellChat version 1.6.1	Jin et al.^50^	N/A
Connectome version 1.0.1	Raredon et al.^51^	N/A
GGplot2 version 3.4.0	https://ggplot2.tidyverse.org	N/A
SCpubR version 1.0.4	https://github.com/enblacar/scpubr/	N/A
ProFAT tool	http://ido.helmholtz-muenchen.de/profat	N/A

## Highlights

- scRNA-seq reveals a massive increase of LAM in BAT of obese mice
- Metabolically stressed brown adipocytes release EVs containing mitochondria
- EVs prime LAM phenotype through lipid scavenger CD36
- LAMs drive brown adipocyte loss identity via TGF-β1-Aldh1a1 pathway
