# Supplementary material for: Lipid-associated macrophages reshape BAT cell identity in obesity
Source: Cell Rep. Author manuscript; Available in PMC 2025 Jan 2. (PMC11693933; doi:10.1016/j.celrep.2024.114447)
Supplement: 1 [file NIHMS2041215-supplement-1.pdf]

Cell Reports, Volume *43*

Supplemental information

Lipid-associated macrophages reshape

BAT cell identity in obesity

Francesca Sciarretta, Andrea Ninni, Fabio Zaccaria, Valerio Chiurchiù, Adeline Bertola, Keaton Karlinsey, Wentong Jia, Veronica Ceci, Claudia Di Biagio, Ziyan Xu, Francesco Gaudioso, Flavia Tortolici, Marta Tiberi, Jiabi Zhang, Simone Carotti, Sihem Boudina, Paolo Grumati, Beiyan Zhou, Jonathan R. Brestoff, Stoyan Ivanov, Katia Aquilano, and Daniele Lettieri-Barbato

SUPPLEMENTAL FIGURES

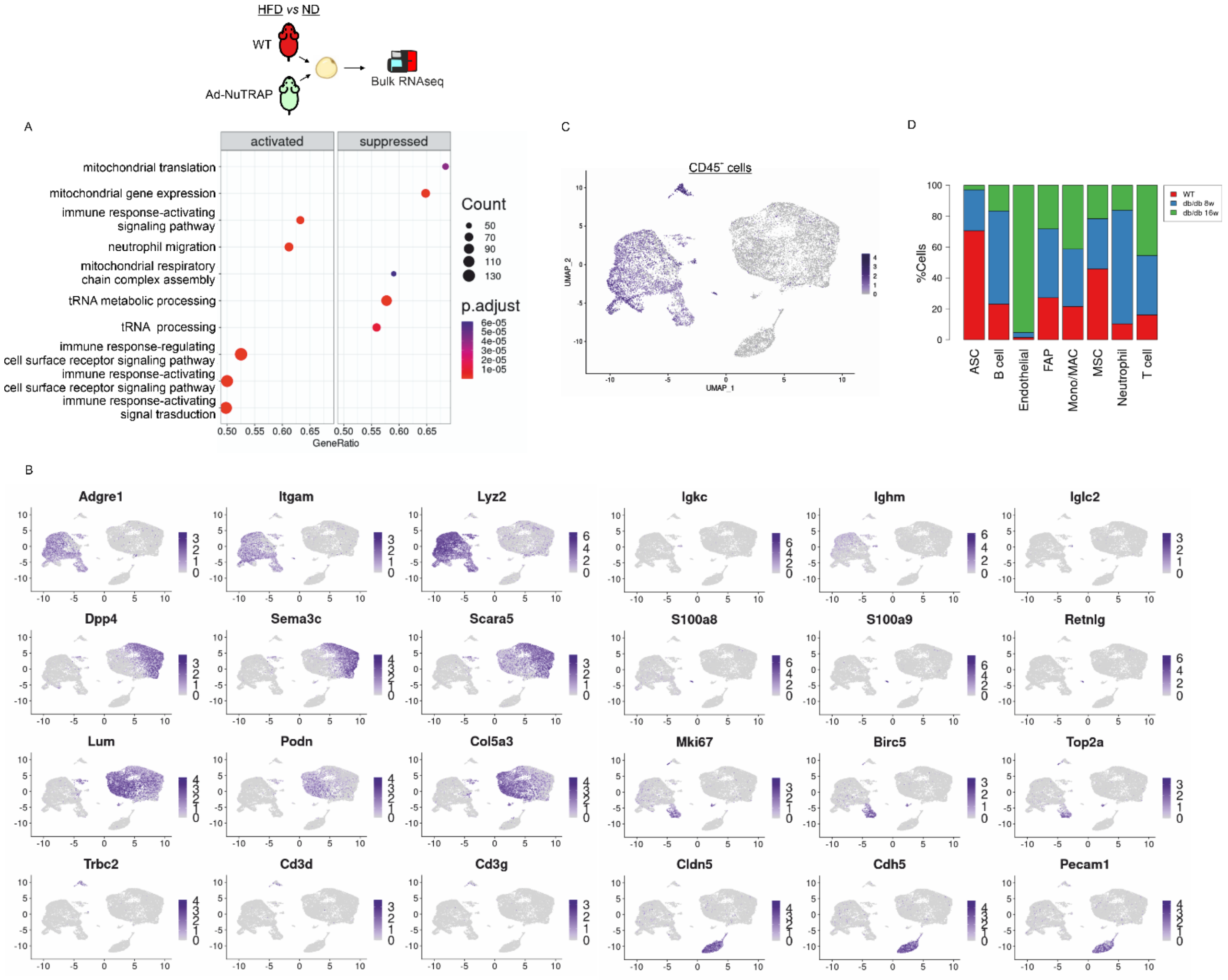

<u>Figure S1</u>. Obesity reduces brown fat genes and increases Mono/MAC in BAT. (A) Gene set enrichment analysis in mature adipocytes isolated from AT of WT and Ad-NuTRAP mice fed with HFD (GSE153120). (**B**) UMAP of genes expression markers identified by single cell RNA-seq of SVFs isolated from BAT of 8 and 16 weeks old wild type (WT) and T2D (db/db) mice (SVF pool from BAT of n=3 mice/group). (**C**) CD45+ cells identified by single cell RNA-seq of SVFs isolated from BAT of 8 and 16 weeks old wild type (WT) and T2D (db/db) mice (SVF pool from BAT of n=3 mice/group). (**D**) Bar plots reporting cell types identified by single cell RNA-seq of the SVFs isolated from BAT of 8 and 16 weeks old wild type (WT) and T2D (db/db) mice (SVF pool from n=3 mice/group).

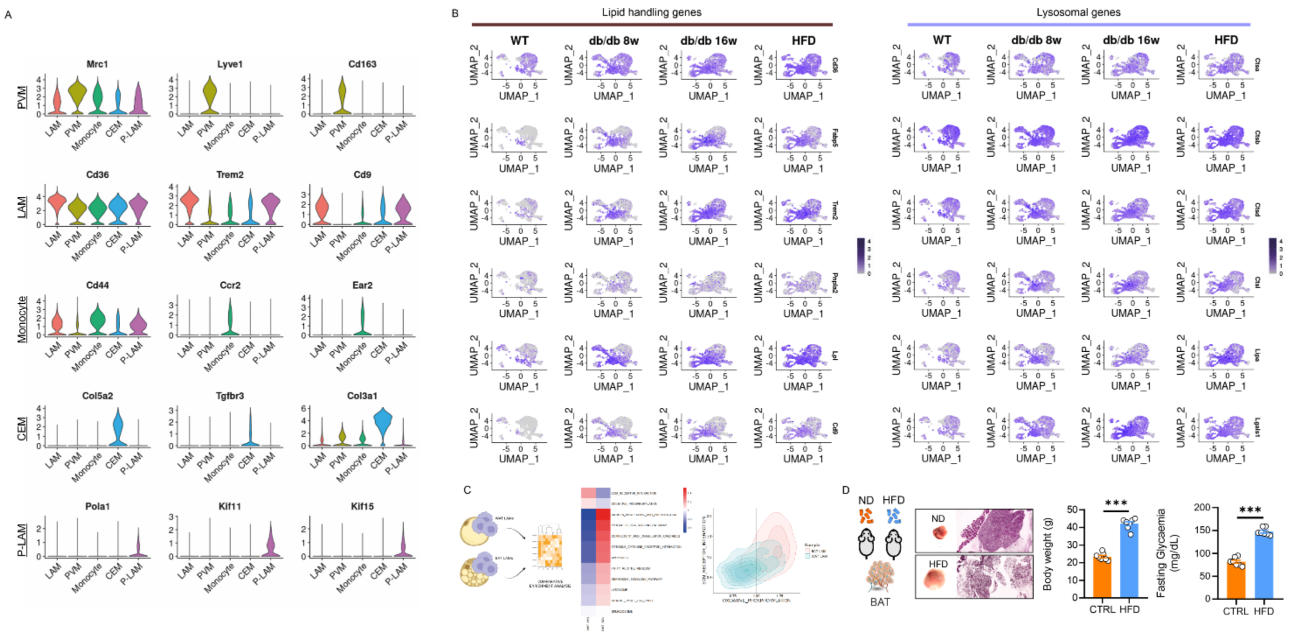

<u>Figure S2</u>. Lipid-associated macrophages are increased in BAT of db/db and HFD mice. (**A**) Violin plots reporting gene markers for cell type identified by single cell RNA-seq in the SVFs isolated from BAT of WT, db/db and HFD mice (SVF pool from n=3 mice/group). (**B**) Lipid handling and lysosomal genes in LAM and P-LAM identified by single cell RNA-seq in the SVFs isolated from BAT of WT, db/db and HFD mice (SVF pool from n=3 mice/group). (**C**) Comparative pathway analysis of LAM between brown and white adipose tissue (GSE182233). (**D**) BAT mass, total body weight and fasting glycaemia of mice fed with normal diet (ND) or HFD for 10 weeks (n=7 mice/group. Data were reported as mean ± SD. Student's t test ***p<0.001).

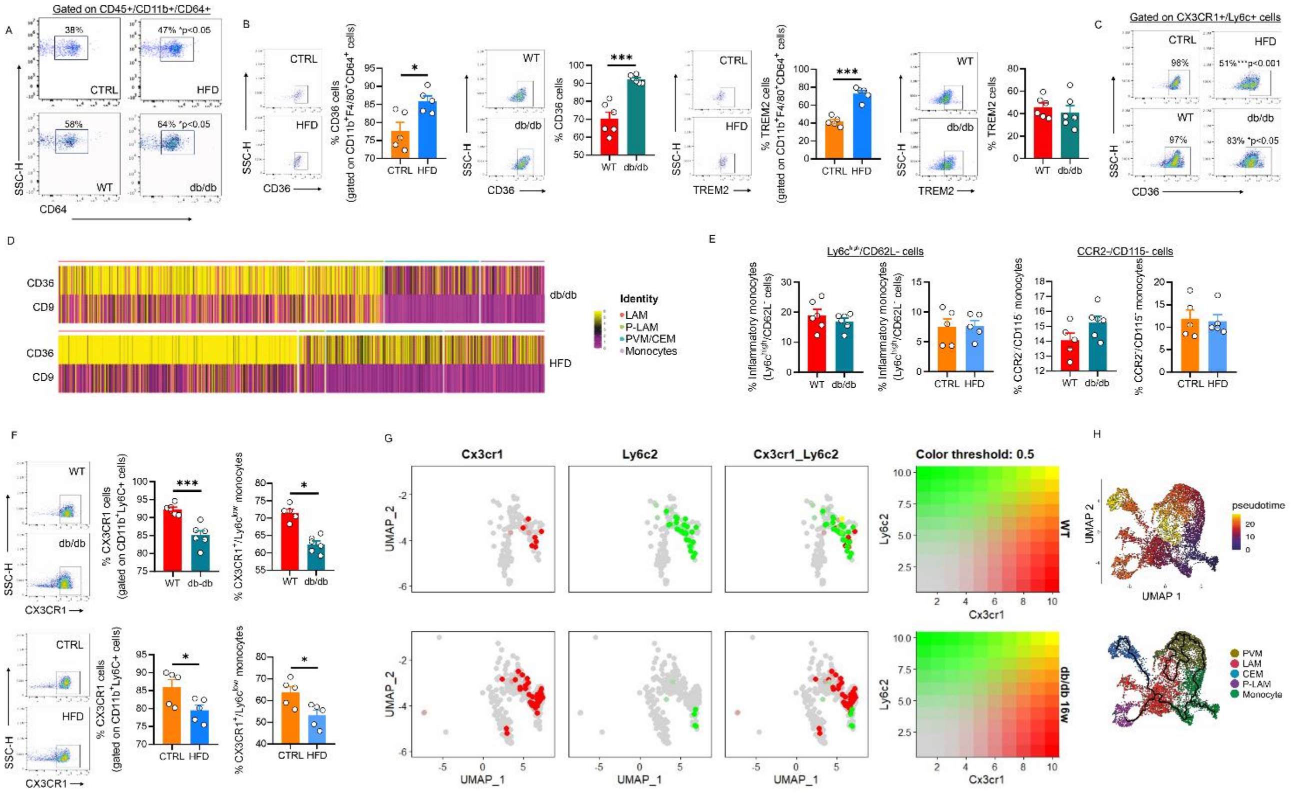

<u>Figure S3</u>. Monocyte/macrophage dynamics in BAT of obese mice. (**A**) Representative flow cytometry analysis of BAT resident macrophages (CD45+/F4.80+/CD64+ cells) of WT, 8-weeks-old db/db and HFD mice (n=5/6 mice/group). Data were reported as mean percentage. Student's t test *p<0.05). (**B**) Flow cytometry measurements of CD36 expression in BAT resident macrophages (CD45+/F4.80+/CD64+ cells) of WT, 8-weeks-old db/db and HFD mice (n=5/6 mice/group). Data were reported as mean percentage. Student's t test *p<0.05; ***p<0.001). (**C**) Representative flow cytometry analysis of CD36 expression in BAT inflammatory monocytes (CD45+/CX3CR1+/Ly6chigh cells) of WT, 8-weeks-old db/db and HFD mice (n=5/6 mice/group). Data were reported as mean percentage. Student's t test *p<0.05; ***p<0.001). (**D**) Heatmap of CD36 and CD9 expression in monocyte/macrophage cell clusters identified by scRNAseq of BAT isolated from obese mice. (**E-F**) Flow cytometry analysis of monocyte dynamics in BAT of WT, 8-weeks-old db/db and HFD mice (n=5/6 mice/group). Data were reported as mean percentage. Student's t test *p<0.05; ***p<0.001). (**G**) Density plot and heatmap reporting CX3CR1 and Ly6c2 expression in monocytes identified in BAT of WT and 8-weeks-old db/db mice by scRNAseq (SVF pool from n=3 mice/group). (**H**) Pseudotime and transcriptional trajectories of monocytes macrophages identified in BAT of WT and 8-weeks-old db/db mice by scRNAseq (SVF pool from n=3 mice/group).

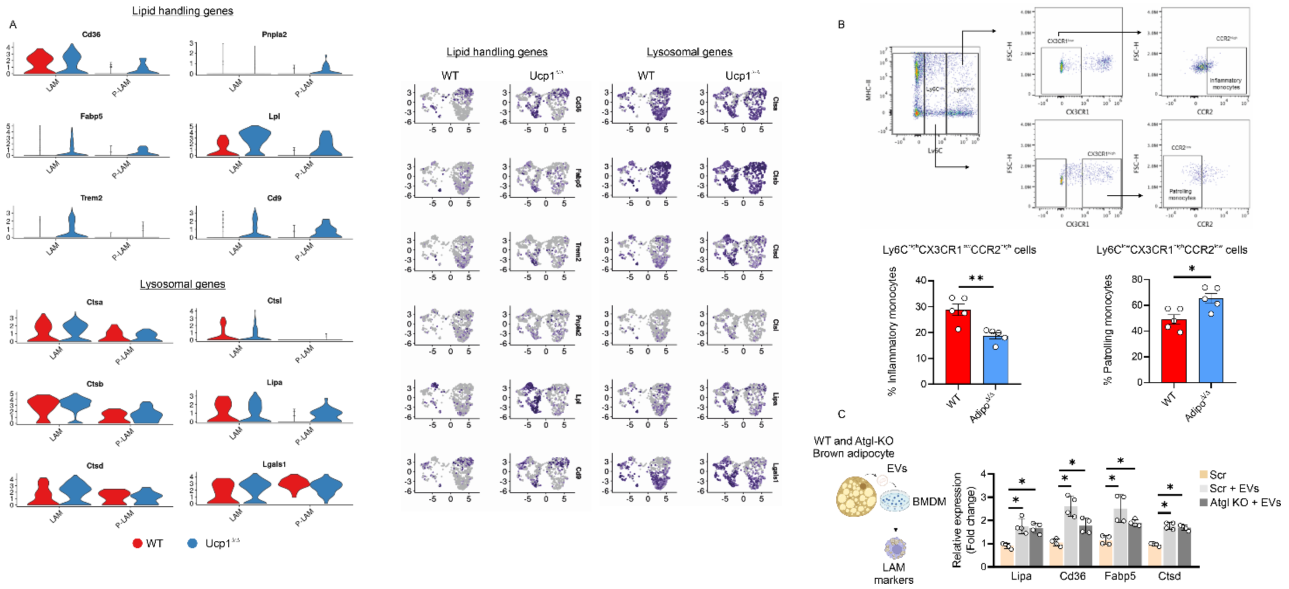

<u>Figure S4</u>. BAT of $Ucp1^{\Delta/\Delta}$ mice shows increased levels of lipid-associated macrophages. (**A**) Violin plot (left panel) and UMAP (right panel) of lysosomal and lipid-handling genes identified by single cell RNA-seq in CD45^{+} cells isolated from BAT of WT and $Ucp1^{\Delta/\Delta}$ mice (n=5 mice/group) (GSE177635). (**B**) Gating strategy (left panel) and flow cytometry measurements of monocytes identified in BAT of WT and $Ucp1^{\Delta/\Delta}$ mice (n=5 mice/group. Data were reported as mean ± SD. Student's t test *p<0.05; **p<0.01). (**C**) Single gene expression levels in bone marrow-derived macrophages (BMDM) treated with EVs released from scr or Atgl downregulating T37i brown adipocytes stimulated with FCCP (10μM for 16h hours in serum-free media). Data were reported as mean ± SD. Student's t test *p<0.05.

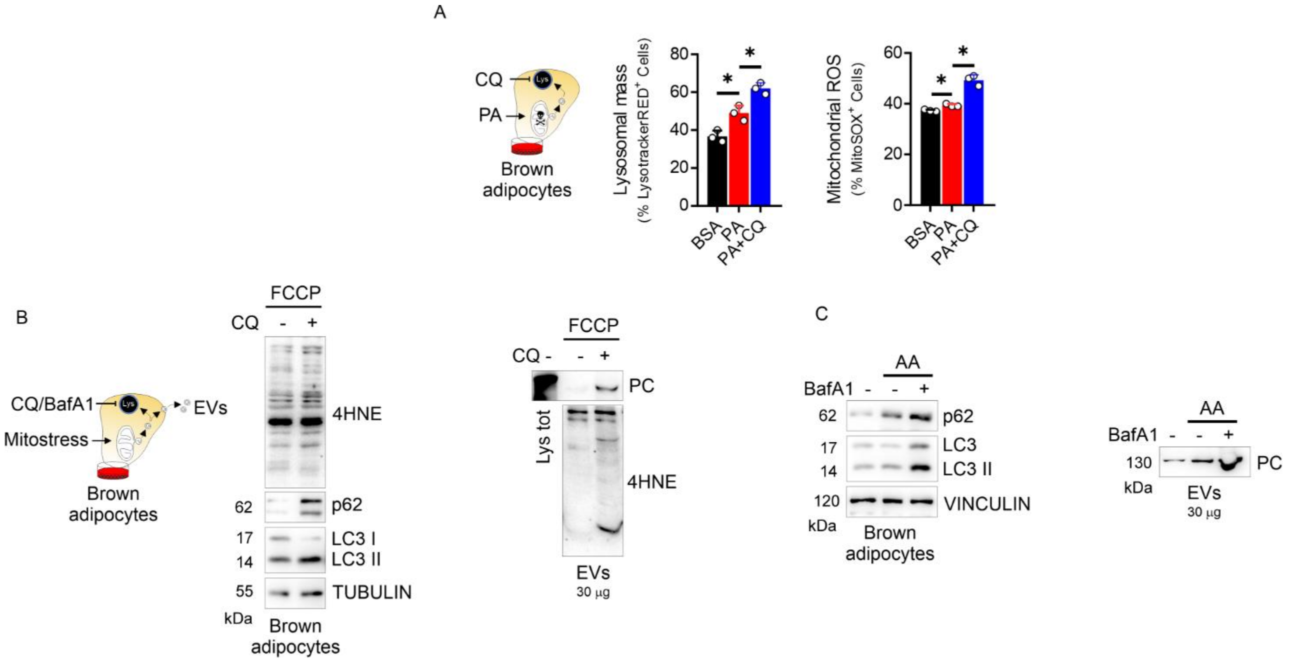

<u>Figure S5</u>. Lysosomal clearance inhibition forces mitochondrial ejection from mature brown adipocytes. (**A**) Flow cytometry measurements of lysosomal mass (*left panel*) and mitochondrial ROS (*right panel*) in mature brown adipocytes treated with palmitate (PA) or chloroquine (CQ). (n=3 independent experiments). Data were reported as mean ± SD. Student's t test *p<0.05. (**B, C**) Representative immunoblots of 4-HNE,

p62, LC3 and PC in cell lysate (left panel) and EVs (right panel) released from brown adipocytes treated with carbonyl cyanide-p-trifluoromethoxyphenylhydrazone (FCCP), antimycin A (AA), chloroquine (CQ) or bafilomycin A1 (BafA1). TUBULIN and VINCULIN were used as loading controls.

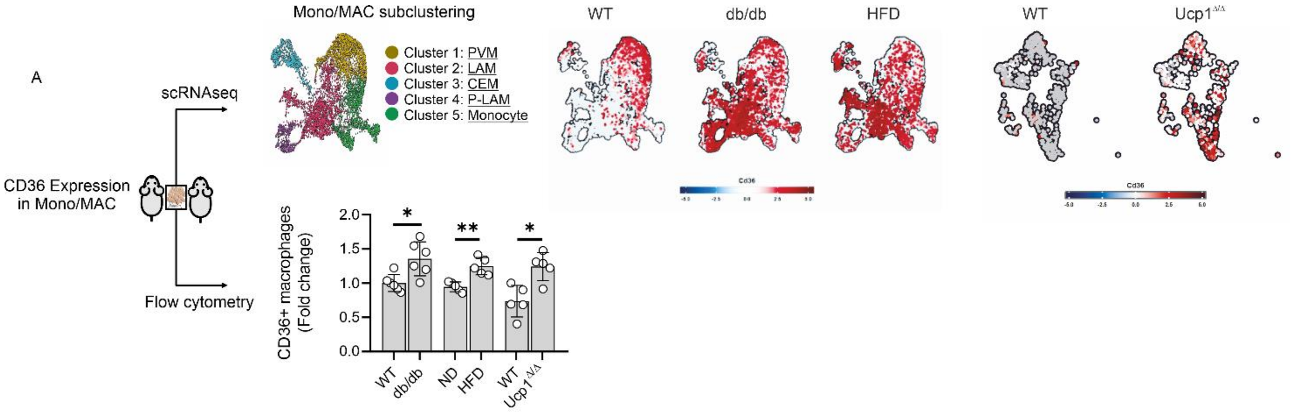

<u>Figure S6</u>. CD36^{+} macrophages are increased in BAT of obese mice. (**A**) CD36+ macrophages identified in BAT of WT, db/db, HFD and Ucp1$^{\Delta/\Delta}$ mice by single cell RNA-seq (*upper panel*) and flow cytometry (*lower panel*). Data were reported as mean ± SD. Student's t test *p<0.05; **p<0.01 (n=5 mice/group).

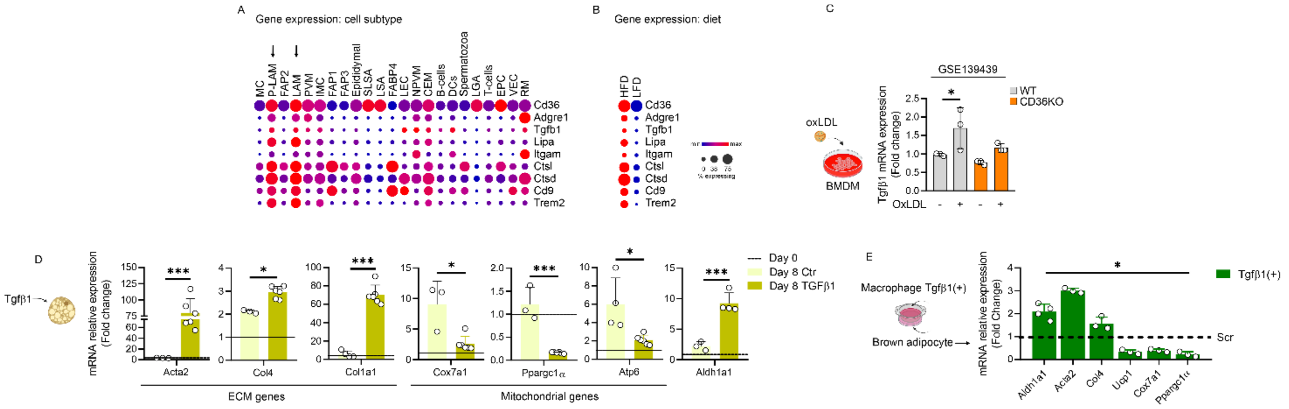

<u>Figure S7</u>. LAM-deriving Tgfβ1 reduces brown fat genes via Aldh1a1. (**A**) Single nuclei data revealing lipid and lysosomal-handling and Tgfβ1 expression in the cell subtypes identified in the visceral adipose tissue of adult mice (SCP1179). (**B**) Single nuclei data revealing lipid and lysosomal-handling and Tgfβ1 expression identified in the visceral adipose tissue of adult mice following low or high fat diet (SCP1179). (**C**) Tgfβ1 expression in BMDM WT and CD36KO treated with oxLDL (GSE139439) Data were reported as mean ± SD. Student's t test *p<0.05. (**D**) Expression level of genes of ECM, mitochondria and Aldh1a1 in brown adipocytes treated with TGFβ1 (10ng/mL) for 16 hours. Data were reported as mean ± SD. Student's t test *p<0.05; ***p<0.001. (**E**) Single gene expression levels of brown fat and ECM genes in primary brown adipocytes co-coltured with RAW264.7 macrophages overexpressing Tgfβ1. Data were reported as mean ± SD. Student's t test *p<0.05.

Table 4. **RT-qPCR Primers List**

	FOWARD	REVERSE
Adgre1	FWD 5’-CCAGGAGTGGAATGTCAAGATGT-3’	REV 5’-GCAGACTGAGTTAGGACCACA-3’
Cd36	FWD 5’-AAGGCCATCTCTACCATGCC-3’;	REV 5’-TGTGGCTAAATGAGACTGGGAC-3’
Rpl8	FWD 5’-GGAGCGACACGGCTACATTA-3’	REV 5’-CCGATATTCAGCTGGGCCTT-3’
PGC1-Alpha	FWD 5’-CTGGAACTGCAGGCCTAACT-3’	REV 5’-GCAAGAGGGCTTCAGCTTTG-3’
Tnf-Alpha	FWD 5′-ATGGCCTCCTCATCAGTT C-3’	REV 5′-TTGGTTTGCTACGACGTG-3’
Aldh1a1	FWD 5’-TCAGGAGTTTACATCAACTGGGA-3’	REV 5’-ACCCCAAACTCCAACCAAGA-3’
TGF1-Beta	FWD 5’-ACCGCAACAACGCCATCTAT-3’	REV 5’-TGCTTCCCGAATGTCTGACG-3’
Pparg	FWD 5’-AGAAAACCAAGGGACCCGAAAT-3’	REV 5’-GTTGGCTTCTTTCAAATCTGGTGT-3’
Atp6	FWD 5’-GCCATTCCACTATGAGCTGGAGCC-3’	REV 5’-GTGGAAGGAAGTGGGCAAGTGAGC-3’
Col4	FWD 5’-GTCCCAGGAATAGGGCCACC-3’	REV 5’-CAGGCATATCCAGTCCGGGG-3’
Col1a1	FWD 5’-GTACATCAGCCCGAACCCCA-3’	REV 5’-GGTGGACATTAGGGCGCAGGA-3’
Acta2	FWD 5’-CAGCCATCTTTCATTGGGATGGA-3’	REV 5’-CCCCCTGACAGGACGTTGTTA-3’
Lipa	FWD 5’-GACCACTCCCGATGCAACTC-3’	REV 5’-GACCGAGTGTTCCTCACCAG-3’
Cidea	FWD 5’-CTCATCAGGCCCCTGACATT-3’	REV 5’-AAGGGCGAGCTGGATGTATG-3’
Cox7a1	FWD 5’-GCTGAGGACGCAAAATGAGG-3’	REV 5’-GTCATTGTCGGCCTGGAAGA-3’

Table 5. **Flow Cytometry Antibody List**

**Antibody**	**Source**	**Dilution**	**Identifier**
CD11b Antibody, anti-human/mouse, APC-Vio 770	Miltenyi Biotec	1:80	Cat# 130-113-232; RRID:AB_2726043
Brilliant Violet 510 anti-mouse CD45 antibody	Biolegend	1:100	Cat#103138; RRID:AB_2563061
APC anti-mouse F4/80 antibody	Biolegend	1:100	Cat#123116; RRID:AB_893481
APC/Cyanine7 anti-mouse F4/80 antibody	Biolegend	1:100	Cat# 123117; RRID:AB_893489
PE anti-mouse CD115 antibody	Biolegend	1:100	Cat#135505; RRID:AB_1937254
PerCP/Cyanine5.5 anti-mouse CD36 antibody	Biolegend	1:100	Cat#102619; RRID:AB_2750187
Brilliant Violet 650anti-mouse CD62L	Biolegend	1:100	Cat#104453; RRID:AB_2800559
Brilliant Violet 711anti-mouse CD64	Biolegend	1:100	Cat#139311; RRID:AB_2563846
FITC anti-mouse CD192 (CCR2) antibody	Biolegend	1:100	Cat#150608; RRID:AB_2616980
PE/Cyanine7 anti-mouse Ly-6C antibody	Biolegend	1:100	Cat#128018; RRID:AB_1732082
APC anti-mouse CD11c antibody	Miltenyi Biotec	1:80	Cat#130-102-800; RRID:AB_2660155
MHC Class II Antibody, anti-mouse, PE-Vio770	Miltenyi Biotec	1:100	Cat# 130-112-232; RRID:AB_2652915
Brilliant Violet 421 anti-mouse CX3CR1 antibody	Biolegend	1:100	Cat#149023; RRID:AB_2565706
Rat Anti-Mouse Trem-2 Monoclonal antibody	R&D	1:100	Cat#FAB17291A; RRID:AB_884527
PerCP/Cyanine5.5 anti-mouse CD9 Antibody	Biolegend	1:100	Cat#124817; RRID:AB_2783076
BUV615 anti-mouse CD80 (B7-1) antibody	BD Biosciences	1:200	Cat# 751328; RRID:AB_2875337
Brilliant Violet 421 anti-mouse Ly-6C antibody	BioLegend	1:200	Cat# 128032; RRID:AB_2562178

Brilliant Violet 750 anti-mouse/human CD11b antibody	BioLegend	1:300	Cat# 101267; RRID:AB_2810328
Brilliant Violet 650 anti-mouse F4/80 antibody	BioLegend	1:200	Cat# 123149; RRID:AB_2564589
CD9 monoclonal antibody, PE	eBioscience	1:200	Cat# 12-0091-83; RRID:AB_891496
PE/Cyanine5 anti-mouse CD11c antibody	BioLegend	1:300	Cat# 117316; RRID:AB_493566
PE/Cyanine7 anti-mouse CD36 antibody	BioLegend	1:200	Cat# 102615; RRID:AB_2566121
PE/Fire 810 anti-mouse I-A/I-E antibody	BioLegend	1:200	Cat# 107667; RRID:AB_2894690
Mer Antibody, anti-mouse, REAfinity	Miltenyi Biotec	1:50	Cat# 130-128-215; RRID:AB_2905327
Alexa Fluor 700 anti-mouse CD206 (MMR) antibody	BioLegend	1:200	Cat# 141734; RRID:AB_2629637
APC/Fire 750 anti-mouse CD192 (CCR2) antibody	BioLegend	1:200	Cat# 150630; RRID:AB_2810417
APC/Fire 810 anti-mouse CD45 antibody	BioLegend	1:200	Cat# 103174; RRID:AB_2860600
